# Structure of pre-miR-31 reveals an active role in Dicer–TRBP complex processing

**DOI:** 10.1073/pnas.2300527120

**Published:** 2023-09-19

**Authors:** Sicong Ma, Anita Kotar, Ian Hall, Scott Grote, Silvi Rouskin, Sarah C. Keane

**Affiliations:** ^a^Biophysics Program, University of Michigan, Ann Arbor, MI 48109; ^b^Department of Chemistry, University of Michigan, Ann Arbor, MI 48109; ^c^Department of Microbiology, Harvard Medical School, Boston, MA 02115

**Keywords:** RNA, NMR spectroscopy, microRNA, structural biology

## Abstract

As an essential posttranscriptional regulator of gene expression, microRNA (miRNA) levels must be strictly maintained. The biogenesis of many miRNAs is mediated by trans-acting protein partners through a variety of mechanisms, including remodeling of the RNA structure. miR-31 functions as an oncogene in numerous cancers, and interestingly, its biogenesis is not known to be regulated by protein-binding partners. Therefore, the intrinsic structural properties of the precursor element of miR-31 (pre-miR-31) can provide a mechanism by which its biogenesis is regulated. We determined the solution structure of pre-miR-31 to investigate the role of distinct structural elements in regulating processing by the Dicer–TRBP complex. We found that the presence or absence of mismatches within the helical stem does not strongly influence Dicer–TRBP processing of the pre-miRNAs. However, both the apical loop size and structure at the Dicing site are key elements for discrimination by the Dicer–TRBP complex. Interestingly, our NMR-derived structure reveals the presence of a triplet of base pairs that link the Dicer cleavage site and the apical loop. Mutational analysis in this region suggests that the stability of the junction region strongly influences processing by the Dicer–TRBP complex. Our results enrich our understanding of the active role that RNA structure plays in regulating miRNA biogenesis, which has direct implications for the control of gene expression.

## Significance

Mature microRNAs, in complex with Argonaute proteins, function to control protein levels in the cell. Mature microRNAs are produced via a series of enzymatic processing events from longer primary and precursor transcripts. While proteins are commonly regulators of various steps in the microRNA biogenesis pathway, no proteins that interact with pre-miR-31 have been identified. Therefore, the mechanism by which miR-31 levels are controlled has until now remained elusive. The present study reveals the three-dimensional structure of pre-miR-31 and uncovers a mechanism by which processing by the Dicer–TRBP complex is regulated internally by the pre-miR-31 structure. Insights into the structure and molecular determinants of microRNA biogenesis have implications in RNA-targeted drug development and design of short hairpin RNAs for gene silencing.

MicroRNAs (miRNAs) regulate protein gene expression posttranscriptionally through base pairing with target messenger (m) RNAs to trigger mRNA degradation or translational suppression (1–4). In the nucleus, RNA polymerase II transcribes primary microRNAs (pri-miRNAs) which are subsequently processed by Microprocessor, a protein complex of Drosha and DiGeorge syndrome critical region 8 (DGCR8). Pri-miRNA processing generates precursor microRNAs (pre-miRNAs) that are exported from the nucleus to the cytoplasm for further processing by Dicer, which functions in complex with transactivation response element RNA-binding protein (TRBP) (5–7). Dicer–TRBP processing results in the production of 21-22 nucleotide (nt) mature miRNA duplexes (4, 8). Mature miRNAs function in concert with Argonaute (Ago) protein to form the miRNA-induced silencing complex, that is responsible for mRNA degradation or translational suppression (2, 4).

Distinctive regulatory elements for pri-miRNAs and pre-miRNAs have been discovered over the past several decades. These elements include specific sequences within the pri-miRNAs and pre-miRNAs that recruit regulatory proteins (9–12). While protein-mediated regulation is indeed important for many pre-miRNAs (13, 14), structural features of pri- and pre-miRNAs can also mediate enzymatic processing in the absence of protein binding (15–21). Therefore, the intrinsic structural properties of pri- and pre-miRNAs may serve as an alternative mechanism for the regulation of their biogenesis, suggesting that the RNA is not a passive element in the miRNA biogenesis pathway.

MicroRNA-31 (miR-31) acts as oncogene in multiple cancers. Upregulation of miR-31 in cells is associated with cancer proliferation, anti-apoptosis, and migration in multiple cancers by targeting different pathways (22). Interestingly, no protein binding partners have been identified for pre-miR-31 (23), suggesting that the mechanisms for regulating miR-31 biogenesis may be encoded at the RNA level. We sought to examine the RNA structural features that may contribute to the posttranscriptional regulation of pre-miR-31.

Here, we describe the three-dimensional structure of pre-miR-31 and characterized how secondary structural elements throughout pre-miR-31 affect Dicer–TRBP processing. The structure presented in this work is the first full-length pre-miRNA structure determined and significantly adds to the limited known structures of pre-miRNAs (24, 25). We found that modulating the structure of pre-miR-31 at the dicing site by enlarging the internal loop reduced the rate of Dicer–TRBP processing. Furthermore, we demonstrate that pre-miR-31 RNAs with extended junction regions, which restrict the apical loop size, displayed significantly reduced processing. However, pre-miR-31 constructs with large apical loops had near wild type (WT)-like levels of processing. Interestingly, in the absence of TRBP, pre-miR-31 RNAs with large apical loops were poor substrates for Dicer processing. These results suggest that the loop size must be tightly controlled, as too small of an apical loop can inhibit pre-miR-31 maturation, a restriction that can be overcome to some extent by the addition of TRBP.

Finally, we found that the junction region functions exquisitely to maximize efficient processing. We note differences in the secondary structure models derived from NMR spectroscopy and chemical probing in the junction region. Rather than viewing these structures as incompatible, we demonstrate that both structures likely exist in a dynamic equilibrium where the base-paired junction transiently samples the open conformation. Our data are consistent with a model in which RNAs can self-regulate their processing in the absence of trans-acting RNA-binding proteins. Recent studies demonstrate the importance of pre-miRNA structural plasticity in regulating their enzymatic processing (20, 21). Our research cements the hypothesis that pre-miRNA structure can regulate its maturation process and further informs on structural features necessary for effective short hairpin (sh) RNA design.

## Results

### The Secondary Structure of FL-pre-miR-31 Contains Three Mismatches in the Helical Stem and Three Base Pairs in the Apical Loop.

**Fig. 1.**
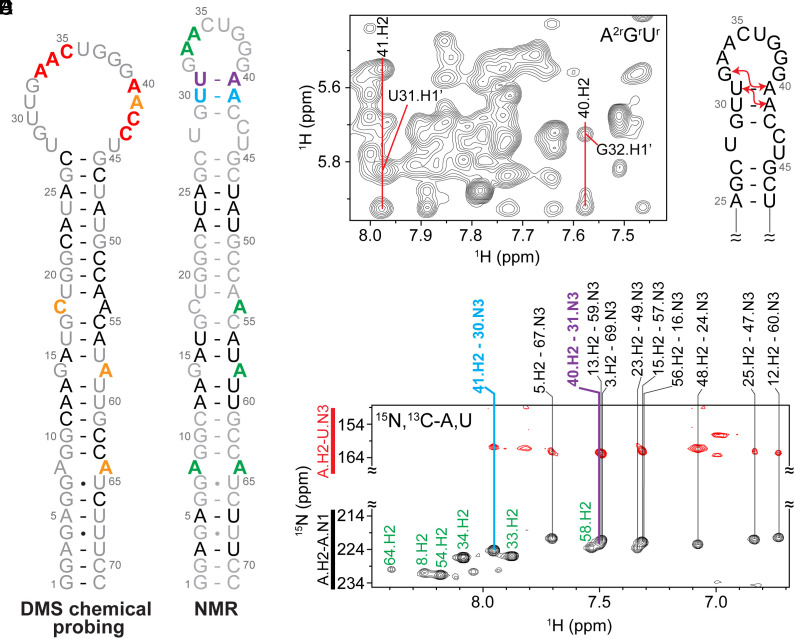
Conflicting secondary structure models for pre-miR-31 apical loop. (*A*) Secondary structure derived from in vitro DMS-MapSeq where coloring denotes reactivity of given bases. Red=high reactivity, orange=medium reactivity, black=low reactivity, gray=no data available. (*B*) Secondary structure derived from NMR characterization. Coloring is based on the identification of A-U base pairs (see panel *E*). (*C*) Portion of a 2D ^1^H–^1^H NOESY spectrum of an A^2r^G^r^U^r^-labeled FL pre-miR-31. Adenosine cross-strand NOEs consistent with helical stacking in the junction region are indicated. (*D*) Secondary structure of the apical loop region highlighting NOEs noted in *C* with red arrows. (*E*) Best-selective long-range HNN-COSY spectrum identifying A-U base pairs within FL pre-miR-31. Black peaks are adenosine H2–N1 correlations, red peaks are adenosine H2–uracil N3 correlations. Vertical lines indicate the detection of A-U base pairs. Unpaired adenosines are denoted in green, A-U base pairs in the stem region are denoted in black, junction A-U base pairs are denoted in cyan and purple.

The lowest free-energy secondary structure of the 71-nt long full-length (FL) pre-miR-31 predicted by the RNAStructure webserver (26) is a hairpin composed of three mismatches (A•A, G•A, and C•A) in the stem region, a 1 × 2 internal loop (dicing site), and three base pairs forming the junction region between the internal and apical loops. However, recent in cell selective 2  ′ hydroxyl acylation analyzed by primer extension (SHAPE) chemical probing studies (27) revealed that the apical loops of pre-miRNAs are less structured than predicted in the miRbase (28–33). To evaluate the secondary structure of FL pre-miR-31, we performed in vitro dimethyl sulfate mutational profiling with sequencing (DMS-MaPseq) (34, 35). The chemical probing derived topology of the entire stem region including the three mismatches is in complete agreement with prediction (Fig. 1*A* and *SI Appendix*, Fig. S1). However, our in vitro chemical probing data suggests that residues within and near the predicted apical loop (A33, A34, C35, A40, A41, C42, and C43) are highly reactive, consistent with these residues being unpaired and forming a large, open apical loop structure (Fig. 1*A* and *SI Appendix*, Fig. S1).

To better understand the molecular details of the pre-miR-31 hairpin, we determined the solution structure of FL pre-miR-31 using NMR spectroscopy. We used a divide-and-conquer approach to facilitate resonance assignments of FL pre-miR-31(*SI Appendix*, Fig. S2). To guide assignments of the FL pre-miR-31 RNA, we combined our previously reported chemical shift assignments for fragments BottomA and BottomB (36) with chemical shift assignments for two additional oligo fragments, TopA (*SI Appendix*, Fig. S3) and Top (*SI Appendix*, Fig. S4). However, the large molecular size of FL pre-miR-31 resulted in a severely crowded spectrum, preventing direct assignments based only on the oligo controls. To better resolve the complex two-dimensional (2D) ^1^H–^1^H NOESY spectrum of FL pre-miR-31, we employed a deuterium-edited approach (*SI Appendix*, Fig. S5) (37–39). The combination of methods allowed for the nearly complete assignment of nonexchangeable aromatic and ribose (C1′, C2′, and C3′) protons in FL pre-miR-31 (*SI Appendix*, Fig. S6 and Table S1).

The NMR-derived secondary structure of FL pre-miR-31 (Fig. 1*B*) is consistent with the predicted lowest free-energy structure. We were particularly interested in the structural features of the apical loop of FL pre-miR-31. Analysis of the ^1^H–^1^H NOESY spectrum of an A^2r^G^r^U^r^-labeled (adenosine C2 and ribose of adenosine, guanosine, and uridine residues are protiated, all other sites deuterated) FL pre-miR-31 revealed strong cross-strand NOEs between A41.H2-U31.H1′ and A40.H2-G32.H1′ (Fig. 1 *C* and *D*), consistent with a typical A-helical structure in this region. To further explore the base pairing within FL pre-miR-31, we acquired a best selective long-range HNN-COSY (40), which allows for the identification of A-U base pairs via detection on the nonexchangeable adenosine C-2 proton rather than the detection of the labile imino proton (*SI Appendix*, Figs. S7 and S8). Here, we see clear evidence for 9 of the 10 expected A-U base pairs within the stem on pre-miR-31 (Fig. 1*E*). The resonance for A53 is broadened beyond detection at pH = 7.5, likely due to the dynamics of the neighboring C18•A54 mismatch. Furthermore, we observe two additional A.H2-U.N3 signals, which correspond to A41-U30 and A40-U31 base pairs (Fig. 1*E*). While A40 and A41 were highly reactive to DMS, and therefore predicted to be unpaired, we provide direct spectroscopic evidence of base pairing within the apical loop.

Consistent with the NMR-derived secondary structure, a pH titration reveals that unpaired residues A8, A54, A64 (mismatches in the helical stem), and A34 (apical loop) are sensitive to the changes in the pH of the solution (*SI Appendix*, Fig. S9). In contrast, the changes of chemical shifts of A40 and A41 (junction) are notably smaller and resemble those measured for base-paired residues from the stem. Additionally, solvent paramagnetic relaxation enhancement (sPRE) data, which reports on the solvent accessibility of FL pre-miR-31, revealed that G29 and A41 do not show large sPRE values (*SI Appendix*, Fig. S10) compared to A33, A34, G37, and G38, which are unpaired in the apical loop. Interestingly, for A40, we observe much higher sPRE value indicating high solvent accessibility of the U31-A40 base pair. These observations suggest that U31-A40 may be a nucleation point for opening the junction based on environmental changes. The sequence of pre-miR-31 is highly conserved in mammals, with mutations or deletions present only in the apical loop region (*SI Appendix*, Fig. S11). Collectively, our results support the presence of a short base-paired element in the junction below the apical loop.

### Tertiary Structure of pre-miR-31.

**Fig. 2.**
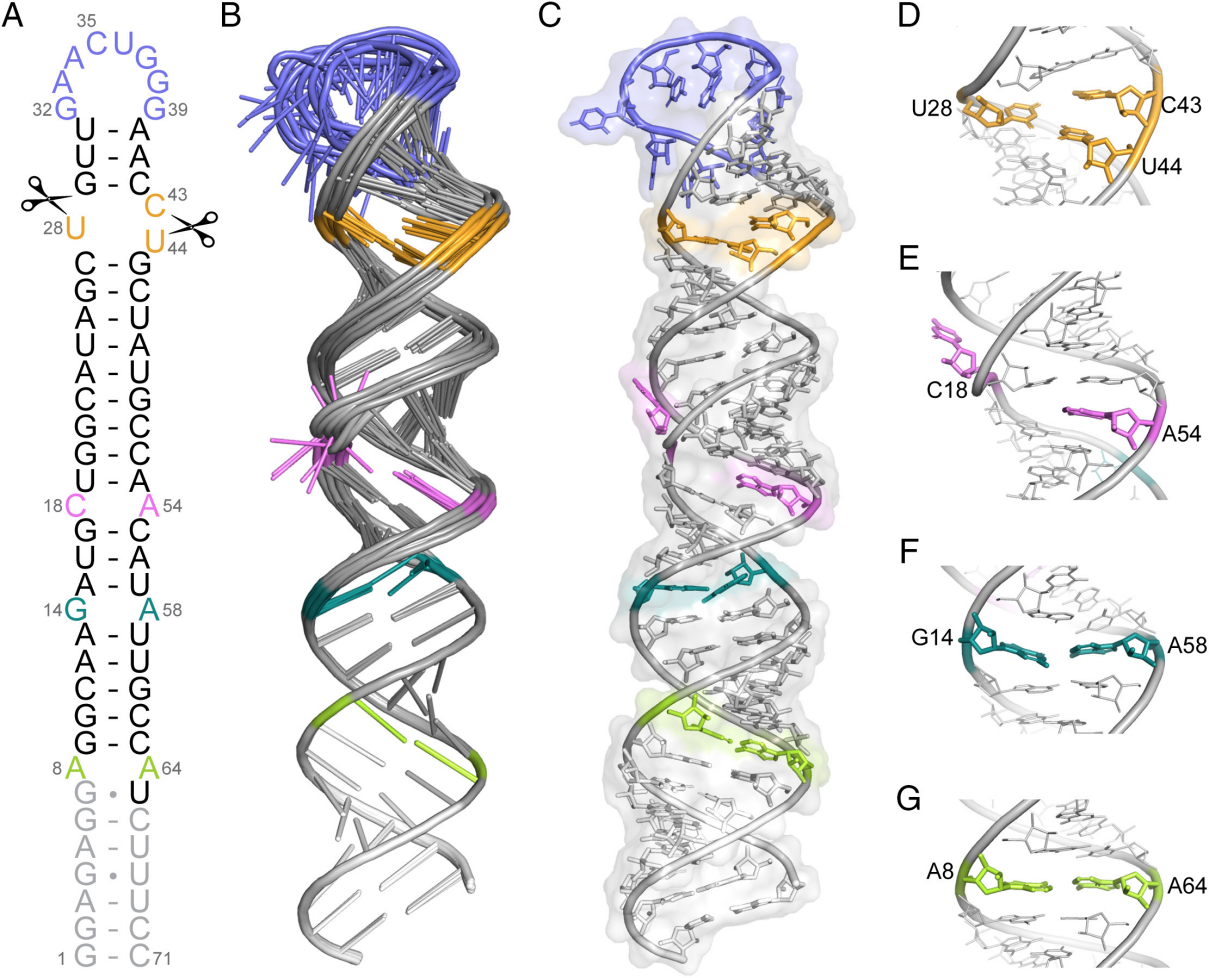
Tertiary structure of pre-miR-31. (*A*) NMR-derived secondary structure of FL-pre-miR-31. Dicer cleavage sites are indicated with scissors. Gray nucleotides were included in structural studies but are not present in a Dicing-competent WT pre-miR-31. (*B*) Ensemble of 10 lowest energy structures after RDC refinement superimposed over residues 1–13 and 59–71. (*C*) The lowest energy structure of pre-miR-31 with a transparent surface rendering. (*D*) Enlarged view of the dicing site, colored orange. (*E*) Enlarged view of the C•A mismatch, colored pink. (*F*) Enlarged view of the G•A mismatch, colored teal. (*G*) Enlarged view of the A•A mismatch, colored green.

To further our structure-based studies, we determined the three-dimensional structure of FL pre-miR-31 (Fig. 2 and *SI Appendix*, Table S2). FL pre-miR-31 adopts a largely elongated hairpin structure, with three base pair mismatches within the helical stem. Nuclear Overhauser effect (NOE) data are consistent with A-helical stacking of 29-GUU-31 and 40-AAC-42, with strong NOEs between A41.H2-U31.H1′ and A40.H2-G32.H1′ (Fig. 1*C* and *SI Appendix*, Fig. S12). The HNN-COSY (Fig. 1*E*) further defines the base pairing within this region, cinching the apical loop structure and limiting the size of the apical loop to 8 nucleotides. The Dicer–TRBP processing site resides within a 1 × 2 internal loop containing U28, C43, and U44 (Fig. 2*D* and *SI Appendix*, Fig. S13). U28 and U44 are coplanar and adopt a *cis* Watson-Crick/Watson-Crick wobble geometry with C43 positioned above U44. We observed a strong NOE between A54.H2 and U19.H1′, which positions A54 stacked in an A-helical geometry (Fig. 2*E* and *SI Appendix*, Fig. S13). No NOEs were observed linking C18 with neighboring residues; therefore, C18 was unrestrained in structure calculations and can sample many conformations (Fig. 2*B*). No defined NOEs were observed connecting A13 with G14. However, aromatic–aromatic and aromatic–anomeric NOEs position G14 stacked under A15. G14 and A58 have the potential to form a cis Watson-Crick/Watson-Crick base pair (Fig. 2*F* and *SI Appendix*, Fig. S13). The A8•A64 mismatch is well defined with sequential and cross-strand NOEs (Fig. 2*G* and *SI Appendix*, Fig. S13). The structure was refined using global residual dipolar coupling (RDC) restraints, and we observed a strong correlation between experimentally determined and back-calculated residual dipolar couplings (*SI Appendix*, Fig. S14). Furthermore, we observe strong agreement between the refined NMR solution structure and scattering data obtained using small-angle X-ray scattering (SAXS) (*SI Appendix*, Fig. S15 and Table S3). Importantly, the pre-miR-31 structure is not sensitive to the nature of the monovalent (*SI Appendix*, Fig. S16) or divalent (*SI Appendix*, Fig. S17) cation in solution (vide infra).

### TRBP Inhibits Dicer Processing of pre-miR-31.

In the cell, TRBP is an important cofactor of Dicer that promotes substrate recognition and processing (5, 7, 41, 42) and also contributes to the accuracy of Dicer processing (5, 43). Interestingly, while addition of TRBP generally stimulated the rate of pre-miRNA processing (6), for pre-miR-31, the addition of TRBP reduced the rate of Dicing (42). We observed a similar ~threefold reduction in the apparent rate constant (*k*_obs_) for pre-miR-31 Dicing in the presence of TRBP (*SI Appendix*, Fig. S18 and Tables S4 and S5).

### Mismatches within the Helical Stem Region have no Impact on Dicer–TRBP Cleavage.

Base pair mismatches are a common feature within the helical stem of pre-miRNAs (36, 44). Studies on fly Dicer-1 suggest that while the length of the pre-miRNA helical stem is important, the presence of mismatches does not significantly affect Dicer processing (16). However, because pre-miR-31 biogenesis does not appear to be regulated by protein binding partners, we considered all aspects of pre-miR-31 structure that could be involved in regulating processing, including individual base pair mismatches.

We first designed a series of mutations that stabilized each mismatch individually and assessed their processing by the Dicer–TRBP complex. Conversion of the G14•A58 mismatch into a canonical U-A base pair (G14U, *SI Appendix*, Fig. S19) exhibits WT rates and levels of processing (*SI Appendix*, Table S4 and Fig. S19). We previously investigated the pH-dependence of the C18•A54 mismatch and found that A54 is partially protonated at physiological pH, suggesting the potential formation of a C•A^+^ base pair near neutral pH (36). We were therefore interested in testing whether mutations that replaced the mismatch with a canonical U-A or C-G base pair (C18U and A54G, respectively) affected the processing by Dicer–TRBP (*SI Appendix*, Fig. S19). As with stabilization of the G•A mismatch, stabilization of the C•A mismatch did not affect the apparent rate of Dicer–TRBP processing or production of mature product (*SI Appendix*, Table S4 and Fig. S19). To fully disrupt the C•A mismatch, we engineered an A•A mismatch at this position (C18A, *SI Appendix*, Fig. S19). This mutant behaved similarly to other stem-stabilized mutations (*SI Appendix*, Table S4 and Fig. S19).

All pre-miR-31 stem mutant RNAs were cleaved to a similar extent (approximately 55%) and with a similar apparent rate constant (*SI Appendix*, Table S4), consistent with studies on fly Dicer-1 (16). As described above, the addition of TRBP reduces the pre-miR-31 processing by Dicer (*SI Appendix*, Fig. S18). Therefore, we examined the processing of stem-mutations with Dicer alone, to discern the role of TRBP for substrate recognition. We found that the four individual stem mutants (C18A, C18U, A54G, and G14U) were processed similarly to WT-pre-miR-31 (*SI Appendix*, Fig. S20 and Table S5), consistent with our findings with the Dicer–TRBP complex. Additionally, we examined the Dicer processing of a fully base-paired double mutant (G14U/A54G) that stabilized both mismatches with canonical base pairs. This fully base-paired pre-miRNA was processed similarly to WT-pre-miR-31 (*SI Appendix*, Fig. S20 and Table S5). To determine whether the context of the C•A mismatch was important for Dicing, we swapped the bases (18ACsw) to create an A•C mismatch. Again, we observed no significant change in Dicer processing (*SI Appendix*, Fig. S20).

Collectively, the processing of pre-miR-31 stem mutants was reduced by the addition of TRBP, consistent with our observations with WT-pre-miR-31 (*SI Appendix*, Fig. S18). However, the stem mutations have no effect on Dicer (*SI Appendix*, Fig. S20 and Table S5) or Dicer–TRBP (*SI Appendix*, Fig. S19 and Table S4) processing, relative to WT.

### Structure at the Cleavage Site Affects Dicer–TRBP Processing.

The RNase III and helicase domains of Dicer interact with the upper stem loop region (which includes the apical loop and the dicing site) and studies indicate that the structure in this region may regulate Dicer processing (15, 16, 18, 45, 46). To differentiate the importance of structure at distinct elements within the upper stem loop region, we employed a mutational approach which reshaped the apical loop and the dicing site, independently.

**Fig. 3.**
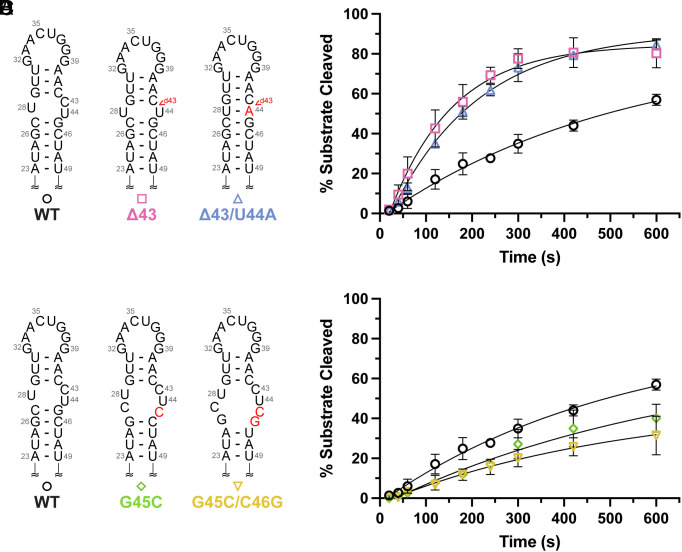
Structure at the dicing site serves as an important feature for Dicer-TRBP processing. (*A*) Predicted secondary structures of constructs designed to minimize the internal loop at the dicing site. Mutations are indicated with red lettering. (*B*) Minimization of the internal loop at the Dicing site enhances the processing by the Dicer–TRBP complex. (*C*) Secondary structures of dicing site mutants with expanded internal loop structures. Mutations are indicated with red lettering. (*D*) Pre-miR-31 RNAs with larger internal loops at the Dicer cleavage site have reduced Dicer-TRBP processing efficiencies, relative to WT. For all processing assays, average and SD from *n* = 3 independent assays are presented.

We generated four different Dicer processing site mutants and examined the impact of structure at this site on Dicer–TRBP processing. Mutations that either minimized (Δ43) or eliminated (Δ43/U44A) the internal loop at the pre-miR-31 Dicer processing site (Fig. 3*A*) displayed significantly enhanced processing (Fig. 3*B* and *SI Appendix*, Table S4). Additionally, mutations that destabilized base pairs proximal to the dicing site (Fig. 3*C*) show a significant reduction in the rate of Dicer–TRBP processing relative to WT (Fig. 3*D* and *SI Appendix*, Table S4).

While the addition of TRBP reduces WT-pre-miR-31 processing, the Δ43C and Δ43C/U44A RNAs exhibited similar processing behavior in both Dicer–TRBP (Fig. 3 *A* and *B* and *SI Appendix*, Table S4) and Dicer-only conditions (*SI Appendix*, Fig. S21 *A* and *B* and Table S5). These results indicate that the Dicer–TRBP complex prefers a smaller (or lack of) internal loop structure at the dicing site. Although constructs with a relatively larger internal loop structure at the dicing site are significantly inhibited in a Dicer-only assay (*SI Appendix*, Fig. S21 *C* and *D* and Table S5), we observe some recovery of activity in the presence of TRBP (Fig. 3 *C* and *D* and *SI Appendix*, Table S4).

### Size and Relative Position of the Apical Loop Regulates Dicer–TRBP Processing.

**Fig. 4.**
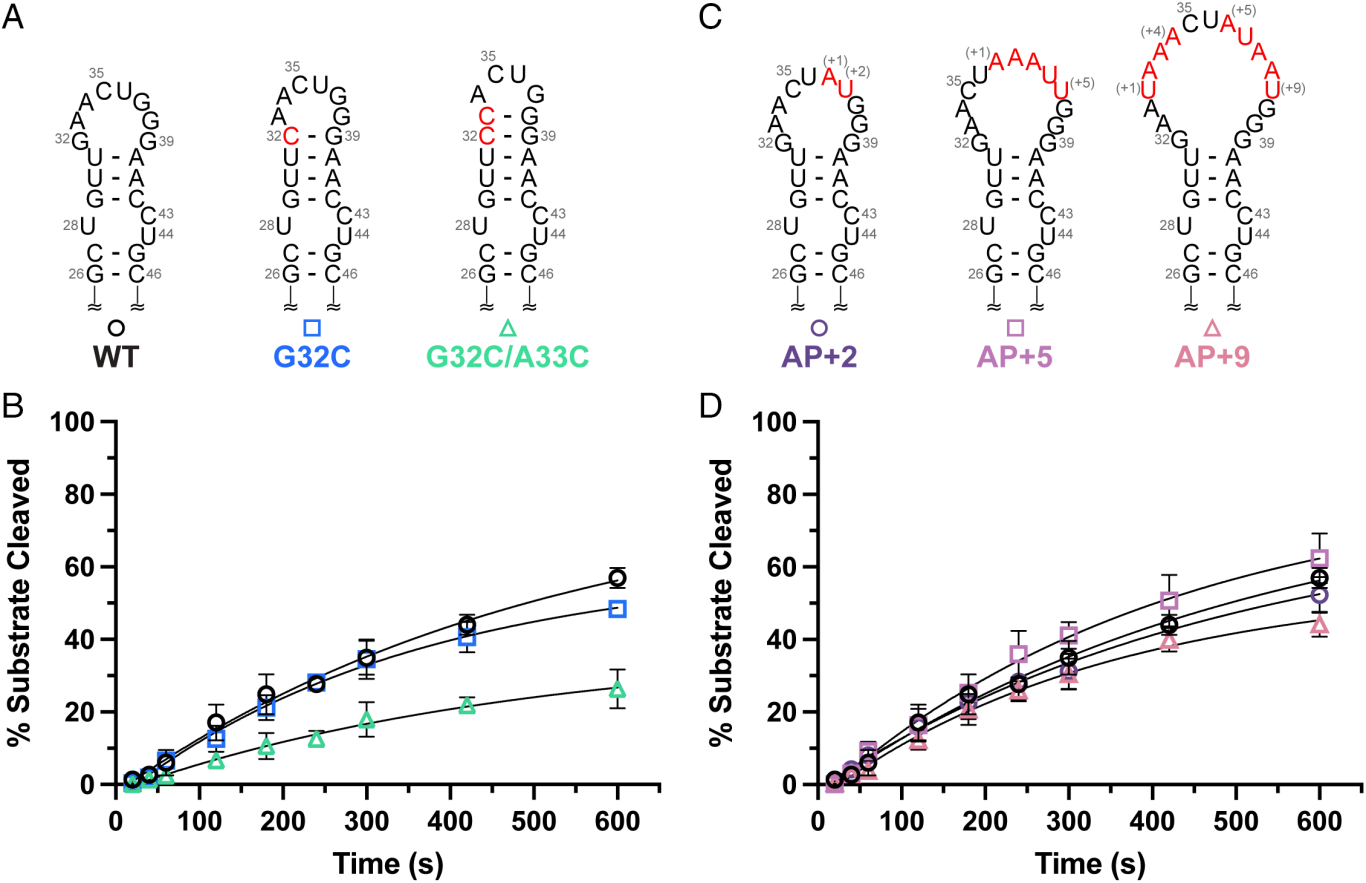
Pre-miR-31 requires a greater than 4-nt apical loop for efficient processing. (*A*) Secondary structures of pre-miR-31 RNAs engineered to contain smaller apical loops. Sites of mutation are denoted with red lettering. (*B*) In vitro processing assays with the Dicer–TRBP complex reveal a significant reduction in substrate cleavage for the G32C/A33C RNA. (*C*) Secondary structures of mutants designed to extend the pre-miR-31 apical loop size. Nonnative nucleotide insertions are indicated with red lettering. (*D*) Dicer–TRBP processing of pre-miR-31 RNAs with larger apical loops was largely unchanged relative to WT. For all processing assays, average and SD from *n* = 3 independent assays are presented.

Apical loop flexibility serves as a control mechanism in many pri/pre-miRNA elements (47, 48), and the apical loop has been identified as a target for regulation by proteins, peptides, and small molecules (13, 49–52). Studies examining the role of apical loop size in Fly Dicer-1 processing revealed that a pre-let-7 RNA with a 4-nt loop were processed less efficiently than a pre-let-7 RNA with a 14-nt loop (16). To build on these findings, we designed two constructs, G32C and G32C/A33C, which minimize the apical loop size by extending the base-paired junction region (Fig. 4*A*). We find that the rate of Dicer–TRBP processing of a G32C/A33C mutant, which restricts the pre-miR-31 apical loop to 4-nt, is reduced by ~2.5-fold (Fig. 4*B* and *SI Appendix*, Table S4). However, the G32C mutant (6-nt apical loop) exhibited WT-like Dicer–TRBP processing rates (Fig. 4*B* and *SI Appendix*, Table S4).

Pre-miRNAs with small apical loops (3–9-nt long) were identified as poor substrates for human Dicer processing, while RNAs with lager apical loops were preferred by Dicer and Drosha (53). We incorporated nonnative nucleotides to the apical loop regions of pre-miR-31 to generate AP+2 (10-nt loop), AP+5 (13-nt loop) and AP+9 constructs (17-nt loop) (Fig. 4*C*). These larger loop mutants do not influence Dicer–TRBP processing (Fig. 4*D* and *SI Appendix*, Table S4).

However, Dicer alone is more sensitive to the apical loop size (*SI Appendix*, Fig. S22 and Table S5). In the absence of TRBP, G32C has a *k*_obs_ that is fourfold lower than that of WT (*SI Appendix*, Fig. S22 *A* and *B* and Table S5). Additionally, we found that the processing of AP+2, AP+5, and AP+9 RNAs was inhibited in the Dicer-only processing assays (*SI Appendix*, Fig. S22 *C* and *D* and Table S5).

The reduction in Dicer processing caused by the presence of a larger apical loop can be offset by other factors, including loop position. Previous studies showed that the apical loop or an internal loop 2-nt from cleavage sites could enhance cleavage efficiency of shRNAs (18, 54). Consistent with previous studies, a pre-miR-31 construct containing an 11-nt loop positioned 2 nt from the cleavage site displayed WT-rates of Dicer processing (40UUG, *SI Appendix*, Fig. S23). Collectively, our findings suggest that TRBP plays an important role in tolerating pre-miRNAs with diverse apical loop sizes and that the presence of TRBP can enlarge the selectivity window for Dicer and promote cleavage for moderately small or large apical loop constructs.

### Junction Residues Function as Critical Control Elements for Dicer Processing.

Our NMR-derived structure of FL pre-miR-31 revealed the presence of three base pairs in a junction region between the apical loop and the dicer cleavage site (Fig. 1*B*). However, *in cell* chemical probing studies with a catalytically inactive Dicer revealed that junction residues were highly reactive, suggesting that these base pairs are absent in the presence of Dicer (27). The high reactivity of these nucleotides *in cell* is consistent with our in vitro chemical probing studies (Fig. 1*A*) which suggest that pre-miR-31 has an unpaired junction region. To assess the functional importance of these alternative structures, we designed constructs which stabilized or destabilized the junction residues and examined the impact on processing. The junction stability and structure were assessed by thermal denaturation of these constructs (*SI Appendix*, Fig. S24 and Table S6).

**Fig. 5.**
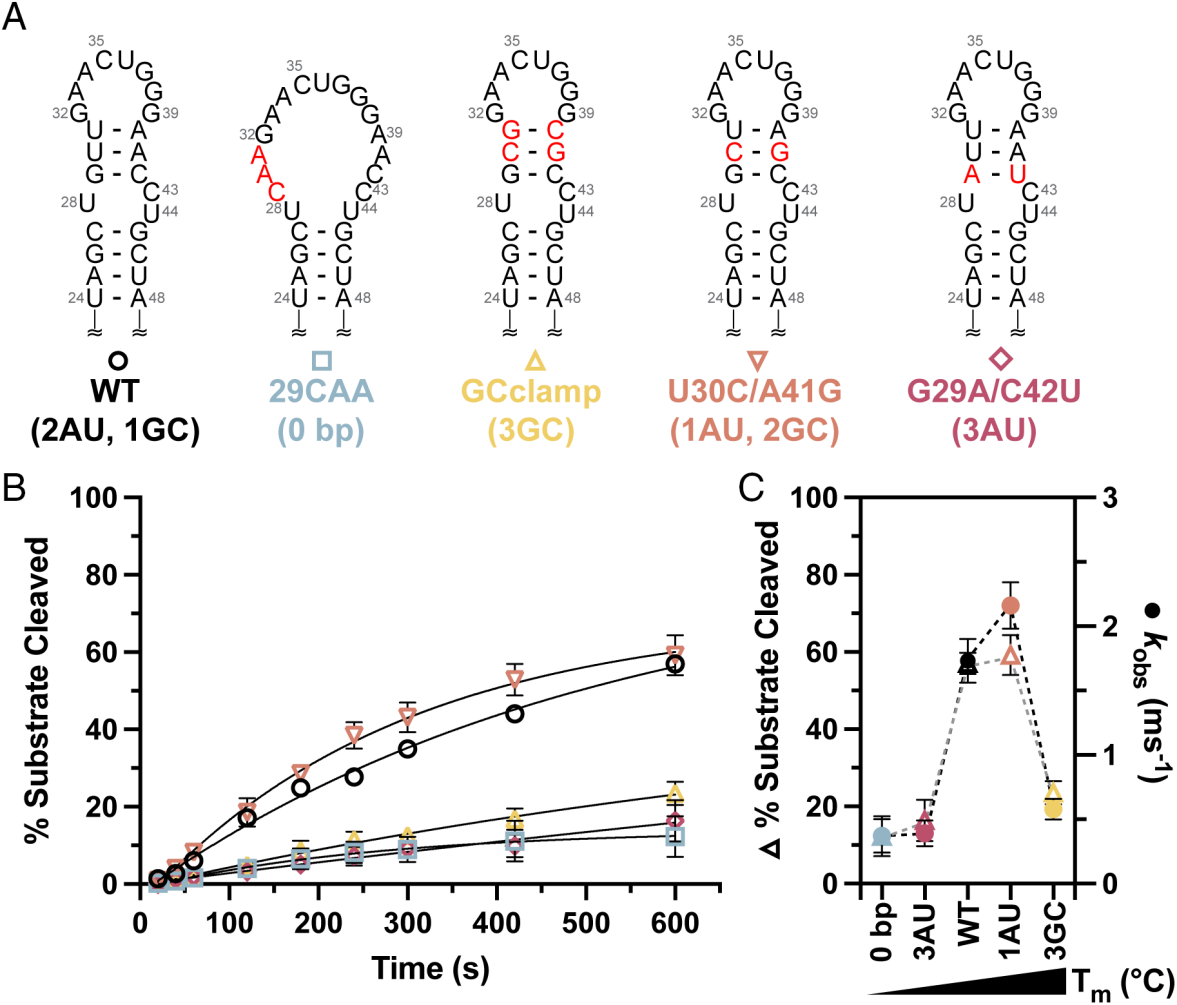
The junction region is a regulatory element within pre-miR-31. (*A*) Design of pre-miR-31 RNAs with varying junction stabilities. Mutations are indicated with red lettering. (*B*) Time-dependent Dicer–TRBP processing of pre-miR-31 junction RNAs reveals the importance of junction stability. (*C*) Correlation between Dicer–TRBP processing (% substrate cleaved and *k*_obs_) and measured thermal stability (melting temperature, *T*_m_) for WT and junction region mutations reveals the need for a moderately stable junction for efficient processing. For all thermal denaturation experiments and processing assays, the average and SD from *n* = 3 independent assays are presented.

To mimic the large open loop structure detected by chemical probing, we mutated residues G29, U30, and U31 to prevent base pairing in the junction region (29CAA) (Fig. 5*A*). The Dicer–TRBP processing data for 29CAA reveals that it is a poor substrate for Dicer–TRBP processing, with a fivefold reduction in k_obs_ for 29CAA relative to WT (Fig. 5*B* and *SI Appendix*, Table S4). We also designed a construct to stabilize the junction region, where the junction A-U base pairs were replaced with G-C base pairs (GCclamp, Fig. 5*A*). Interestingly, the Dicer–TRBP processing of the GCclamp construct was reduced threefold, relative to WT (Fig. 5*B* and *SI Appendix*, Table S4). These data suggest that the stability of the base pairs within the junction region is an important determinant of Dicer–TRBP processing.

To further elucidate how the junction stability of pre-miR-31 regulates Dicer–TRBP processing, we designed two additional junction mutants with different base pairing compositions. The U30C/A41G construct (1AU base pair and 2 GC base pairs, Fig. 5*A*) has a k_obs_ that is 1.25-fold faster than WT (2AU base pairs, 1 GC base pair) (Fig. 5*B* and *SI Appendix*, Table S4). However, the k_obs_ for the G29A/C42U mutant (3 AU base pairs, Fig. 5*A*) is reduced fivefold relative to WT, similar to what we observed for 29CAA (Fig. 5*B* and *SI Appendix*, Table S4). Similar trends hold for the junction mutants in processing assays without TRBP (*SI Appendix*, Fig. S25 and Table S5). These data suggest that the stability of the junction region is finely tuned to maximize Dicer processing.

Collectively, we observe a trend in which pre-miR-31 RNAs with a moderately stable junction (WT, U30C/A41G) are efficiently processed by Dicer–TRBP, suggesting that these structures are ideal substrates. However, pre-miR-31 RNAs with either an over-stabilized junction (GCclamp) or a highly destabilized junction (G29A/C42U and 29CAA) are poor substrates, reflected in their reduced k_obs_ (Fig. 5*C*). This delicate balance of structural stability within the junction must be optimized to maximize efficient processing.

## Discussion

miRNAs play important roles in the posttranscriptional regulation of gene expression in eukaryotes. Due to this important regulatory function, miRNAs are themselves subject to posttranscriptional regulation to ensure that appropriate levels of the mature products are produced. Many proteins are known to posttranscriptionally regulate miRNA biogenesis at either the Drosha and/or Dicer processing steps (4, 12, 13). While protein-mediated regulation of miRNA biogenesis can be an important mechanism of control, the intrinsic structural features of pri/pre-miRNAs can also regulate their enzymatic processing (4, 20, 21). Pre-miR-31 is a pre-miRNA with no identified protein binding partners (23) and was therefore an attractive target for uncovering RNA-mediated mechanisms underlying miRNA biogenesis.

**Fig. 6.**
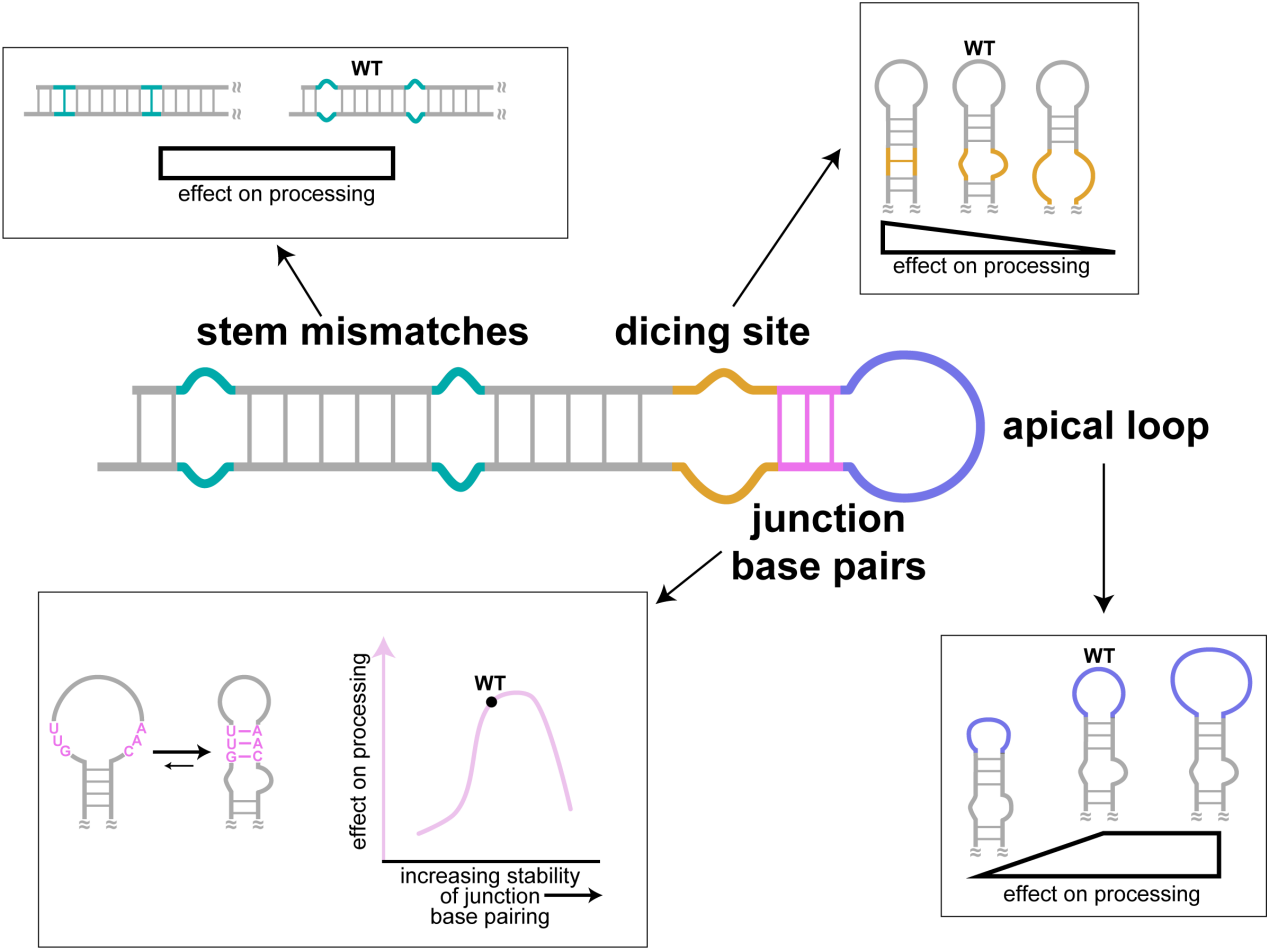
Secondary structure elements and their contribution to the regulation of pre-miR-31 processing. The presence or absence of mismatches within the stem of pre-miR-31 had no impact on Dicer–TRBP processing. RNAs with more stabilized Dicing sites were processed more efficiently than the WT sequence, while pre-miRNAs with larger internal loops were poorly processed. Similarly, pre-miRNAs with too small of an apical loop was processed less efficiently than WT pre-miR-31. Interestingly, the WT pre-miR-31 has an inherently encoded structural switch at the junction region. Pre-miR-31 appears to sample both an open loop structure and a closed loop structure. Only RNAs with marginally stable junction regions were maximally processed by Dicer–TRBP. The ability of pre-miR-31 to sample both states promotes processing of pre-miR-31 by the Dicer–TRBP complex.

To identify the structural basis for Dicer–TRBP processing of pre-miR-31, we solved the high-resolution tertiary structure of the FL pre-miR-31 RNA. Our structural and biochemical studies provide a framework for optimized design of shRNAs and elucidate distinct mechanisms by which RNA structure helps to regulate Dicer–TRBP-mediated processing of pre-miR-31 (Fig. 6).

We found that the presence of mismatches within the pre-miR-31 stem, while a nearly ubiquitous feature of pre-miRNAs, did not significantly influence the processing of pre-miR-31. Furthermore, excluding TRBP did not influence Dicer processing of these mutants. This finding is in contrast to studies with Fly Dicer-1 which demonstrated that mismatches direct the production of different length products in the presence of the Loqs-PB co-protein (44). Previous studies showed the importance of secondary structure at the dicing site for Dicer cleavage of shRNA and some pre-miRNAs (54). Here, we show that RNAs with reduced or eliminated internal loops at the Dicing site are good substrates for Dicer–TRBP processing. However, increasing the internal loop size negatively impacted Dicer–TRBP processing.

Both apical loop size and position contribute to the regulation of Dicer and Drosha processing (15, 16, 18, 53–55). Our findings reemphasized the role of apical loop size on Dicer–TRBP processing and provided additional insights. Previous studies demonstrate that the presence of a small apical loop inhibits Dicer cleavage (16, 53). We showed not only that a small apical loop inhibits Dicer processing but also that large apical loops inhibit Dicer processing. Interestingly, the addition of TRBP appears to widen the apical loop size window of RNAs that are efficiently processed. Our study reveals that loop size is one property that should be optimized when designing shRNAs.

Importantly, we found the junction region of pre-miR-31 to be an inherent regulatory site. Our NMR-derived secondary structure stands in contrast to one revealed by in cell chemical probing (27). Secondary structures reported based on chemical probing adopt a large apical loop region, where the junction residues are unpaired. We believe that the differences in the NMR and chemical probing–derived structures reflect the likely dynamic nature of the base pairs in the junction region, information which can be obstructed in the chemical probing studies. Our structural data are consistent with a model in which some junction base pairs are accessible to solvent and thus more prone to open.

We imagine that the junction region of pre-miR-31 exists in a dynamic equilibrium promoting distinct favorable interactions with the Dicer–TRBP complex. We found that mutations which either stabilized or destabilized the junction region reduced Dicer–TRBP processing. The open apical loop structure sequesters the Dicer cleavage sites in the loop, which may account for the reduced processing levels. Collectively, we found that the stability of the pre-miR-31 junction region is optimized to sample both open and cinched conformations to promote efficient processing. These findings enrich the understanding of how distinct conformations of pre-miR-31 contribute to Dicer–TRBP processing.

Importantly, we note that our structural and enzymatic studies were conducted in vitro under conditions that may not replicate the complex cellular environment. It is indeed possible that regulation in the cell is driven by factors other than pre-miRNA structure and dynamics, for example competition for other pre-miRNAs, varying pre-miRNA expression levels, or crowding factors (56).

Our newly resolved 3D structure of pre-miR-31 in its processing-competent conformation and elucidation of its intrinsic regulatory mechanism informs on the important role that pre-miRNA structural plasticity plays in controlling Dicer processing. Our structural and biochemical studies are consistent with proposed models of pre-miRNA processing based on cryo-EM structures of human Dicer (57) and fly Dicer-1 (55) bound with pre-miRNAs. The pre-let-7 bound human Dicer structure revealed that the pre-let-7 RNA adopts multiple conformations (57). In the “pre-dicing state,” Wang and co-workers posit that the pre-let-7 RNA first binds before the structure is adjusted to form a more stable stem (57). This hypothesis is consistent with our findings that the pre-miR-31 apical loop exists in a conformational equilibrium where a large apical loop structure may be the preferred substrate for Dicer–TRBP binding, but that the structure with a cinched junction region is a “dicing-competent” structure.

The structure of human Dicer bound with let-7a-1^GYM^ was recently reported (46). This structure revealed interactions between the Dicer RNase III a domain and pre-miRNA residues above the dicing site, at the junction region identified in pre-miR-31. This structure further revealed that the pre-miRNA structure was significantly distorted from an A-form helical structure near the cleavage site revealing a need for “flexibility” in this region to be reshaped by Dicer.

The recent cryo-EM structures of fly Dicer-1 reveal further details of the Dicer-1-pre-miRNA structure in the “Dicing” state (55). In the Dicing state, the structure reveals that the pre-miRNA is highly structured, with the Dicing site sequestered in an A-form helical structure and several base pairs present above the Dicing site. This Dicing structure is consistent with our NMR-derived structure, where the stabilization of additional base pairs in the junction promotes the formation of an extended A-helical structure above the dicing site.

Our data suggest that pre-miR-31 is “prestructured” for Dicer processing. The presence of the junction base pairs in pre-miR-31 supports the formation of an A-form helical structure. However, the stability of these base pairs is critical. The base pairing cannot be too tight, or it may inhibit the restructuring of the RNA for binding. Additionally, the structure cannot be too flexible, as the presence of the A-form geometry is important for Dicer–TRBP processing. Further structural studies are necessary to fully characterize the structural changes in both the pre-miRNA and Dicer throughout the catalytic cycle.

## Methods

### Preparation of Recombinant Human Dicer.

Human Dicer protein was purified as previously described (58, 59) with modifications. Sf9 cells with infected His-tagged Dicer baculovirus is purchased from University of Michigan protein core. The cell pellet was lysed in ice-cold lysis buffer (50 mM Na_2_HPO_4_ pH = 8.0, 300 mM NaCl, 0.5% Triton X-100, 5% glycerol, 0.5 mM tris(2-carboxyethyl) phosphine (TCEP) and 10 mM imidazole) by sonication. The lysate was pelleted by centrifugation at 30,000×*g* for 30 min, and the supernatant was mixed with 5 mL preequilibrated Ni-NTA resin (Qiagen) in a 50-mL falcon tube. After gently rocking for 1 h at 4 °C, the resin was pelleted by centrifugation at 183×*g* for 10 min. The resin was washed with 45 mL wash buffer (50 mM Na_2_HPO_4_ pH = 8.0, 300 mM NaCl, 5% glycerol, 0.5 mM TCEP and 20 mM imidazole) five times and eluted with elution buffer (50 mM Na_2_HPO_4_ pH = 8.0, 300 mM NaCl, 5% glycerol, 0.5 mM TCEP and 300 mM imidazole). The elutions were dialyzed against dialysis buffer (20 mM Tris pH = 7.5, 100 mM NaCl, 1 mM MgCl_2_, 0.1% Triton X-100, 50% glycerol). Purified protein was stored at −80 °C and total protein concentration was determined by Bradford assay (Thermo Fisher Scientific), and the concentration of Dicer was quantified using ImageJ.

### Expression and Purification of TRBP.

The expression and purification of human TRBP was based on previously described procedures (60, 61). The pET28a-TRBP was purchased from Addgene (Addgene plasmid # 50351). The pET28a-TRBP plasmid was transformed into *Escherichia coli* Rosetta (DE3) pLysS (Novagen), and cells were grown in LB media until cell density reached OD_600_ = 0.6. Protein expression was induced by the addition of 0.2 mM IPTG and cells were incubated at 18 °C for 20 h. The cells were harvested by centrifugation, resuspend in buffer A (20 mM Tris–HCl pH 8.0, 500 mM NaCl, 25 mM imidazole, and 5 mM β-mercaptoethanol), and lysed by sonication. The lysate was pelleted by centrifugation at 20,000×*g* for 30 min, and the supernatant was loaded onto a nickel affinity column and gradient eluted in buffer B (20 mM Tris–HCl pH 8.0, 500 mM NaCl, 250 mM imidazole buffer, and 5 mM β-mercaptoethanol). The protein elution was collected and 10% polyethyleneimine (PEI) was added dropwise (1.5% vol/vol) to remove nucleic acid contaminants. The suspension was stirred at 4 °C for 30 min and the supernatant was collected by centrifugation (30 min at 20,000 rpm). Two sequential ammonium sulfate cuts were performed (with centrifugation for 30 min at 20,000 rpm in between) at 20% and 80% saturation. The pellet from the 80% ammonium sulfate cut was resuspended in buffer C (20 mM Tris-HCl pH 8.0, 2 mM DTT) and dialyzed against buffer D (20 mM Tris-HCl pH 8.0, 150 mM NaCl, 2 mM DTT) overnight. The overnight dialyzed sample was concentrated and loaded into HiLoad 16/600 Superdex 200 (Cytiva) equilibrated with buffer D.

### Dicer–TRBP Complex Formation.

The purified Dicer and TRBP proteins were mixed at a 1:3 molar ratio and loaded into HiLoad 16/600 Superdex 200 column (Cytiva) equilibrated with buffer D. The fractions enriched with complex were pooled and concentrated to ~534 nM and flash-frozen by liquid nitrogen for use in processing assays.

### Preparation of DNA Templates.

DNA templates for oligo RNAs were purchased from Integrated DNA Technologies (*SI Appendix*, Table S7). The DNA templates for in vitro transcription were created by annealing the DNA oligonucleotides with an oligonucleotide corresponding to the T7 promoter sequence (5′-TAATACGACTCACTATA-3′). Templates were prepared by mixing the desired DNA oligonucleotide (40 µL, 200 µM) with the complementary oligonucleotide to T7 promoter sequence (20 µL, 600 µM) together, boiling for 3 min, and then slowly cooling to room temperature. The annealed template was diluted with water prior to use to produce the partially double-stranded DNA templates at a final concentration approximately 8 µM.

### Preparation of Plasmid Templates for In Vitro Transcription.

The templates for the preparation of the extended pre-miR-31 for DMS-MaPseq and FL pre-miR-31 for NMR studies were generated by overlap-extension (OE) PCR using EconoTaq PLUS 2x Master Mix (Lucigen) with primers listed in *SI Appendix*, Tables S8 and S9. The OE PCR template was digested with EcoRI and BamHI restriction enzymes and inserted into the pUC-19 plasmid. DNA templates for use in in vitro transcription reactions were amplified with EconoTaq PLUS 2x Master Mix (Lucigen) using primers UNIV-pUC19_E105 and miR_tail_3buffer_REV (DMS) or miR31_4R (NMR, *SI Appendix*, Table S10).

To ensure that the native pre-miR-31 used for processing contained homogeneous 5′-AG sequence, of we included a hammerhead (HH) ribozyme 5′ of the pre-miR-31 sequence (62). The native pre-miR-31 template, used to make RNA for processing studies, was generated by OE PCR using EconoTaq PLUS 2x Master Mix (Lucigen) with primers listed in *SI Appendix*, Table S11. The OE PCR template was digested with EcoRI and BamHI restriction enzymes and inserted into pUC-19 plasmid. The HH-pre-miR-31-HDV plasmid, which was designed to ensure a homogeneous 3′ end of the transcript, was generated by inserting HDV ribozyme sequence to 3′ end of HH-pre-miR-31 plasmid construct using the Q5 site-directed mutagenesis kit (New England biolabs) with primers HH-miR-31-HDV-mut-F and HH-miR-31-HDV-mut-R (*SI Appendix*, Table S12). All subsequent mutations, deletions, and/or insertions were achieved via site-directed mutagenesis (New England Biolabs Q5 site-directed mutagenesis kit) of the HH-pre-miR-31-HDV plasmid with primers listed in *SI Appendix*, Table S12. Templates prepared from plasmids were amplified with EconoTaq PLUS 2x Master Mix (Lucigen) using primers UNIV-pUC19_E105 and HDV-AMP-R (*SI Appendix*, Table S10). All primers were purchased from Integrated DNA Technologies. Plasmid identity was verified by Sanger sequencing (Eurofins Genomics) using the universal M13REV sequencing primer.

### Preparation of RNA.

RNAs were prepared by in vitro transcription in 1 × transcription buffer [40 mM Tris base, 5 mM dithiothreitol (DTT), 1 mM spermidine and 0.01% Triton-X (pH = 8.5)] with addition of 3–6 mM ribonucleoside triphosphates (NTPs), 10–20 mM magnesium chloride (MgCl2), 30–40 ng/μL DNA template, 0.2 unit/mL yeast inorganic pyrophosphatase (New England Biolabs) (63), ∼15 μM T7 RNA polymerase, and 10–20% (v/v) dimethyl sulfoxide (DMSO). Reaction mixtures were incubated at 37 °C for 3–4 h, with shaking at 70 rpm, and then quenched using a solution of 7 M urea and 500 mM ethylenediaminetetraacetic acid (EDTA), pH = 8.5. Reactions were boiled for 3 min and then snap-cooled in ice water for 3 min. The transcription mixture was loaded onto preparative-scale 10% denaturing polyacrylamide gels for purification. Target RNAs were visualized by UV shadowing and gel slices with RNA were excised. Gel slices were placed into an elutrap electroelution device (The Gel Company) in 1X TBE buffer. RNA was eluted from the gel at constant voltage (120 V) for ~24 h. The eluted RNA was spin concentrated, washed with 2 M high-purity sodium chloride, and exchanged into water using Amicon-15 Centrifugal Filter Units (Millipore, Sigma). RNA purity was confirmed on 10% analytical denaturing gels. RNA concentration was quantified via UV–Vis absorbance. Sequences for all RNAs is provided in *SI Appendix*, Table S13.

### Dimethyl Sulfate (DMS) Modification of pre-miR-31 RNA.

Three μg of pre-miR-31-tail RNA was denatured at 95 °C for 1 min and incubated on ice for another 3 min. Refolding buffer (300 mM sodium cacodylate and 6 mM MgCl_2_) was added to reach total volume of 97.5 uL (for the 0% control), 97.5 μL (for 2.5% modified sample), or 95 μL (for 5% modified sample). The RNA was incubated in refolding buffer at 37 °C for 40 min. The RNA was treated with either 2.5 µL DMSO (0% DMS), 2.5 μL DMS (2.5% DMS), or 5 μL DMS (5% DMS) followed by incubation at 37 °C while shaking at 250 rpm for 10 min. Then, 60 μL β-mercaptoethanol was added to each reaction to neutralize the residual DMS. The modified RNA was purified using RNA Clean and Concentrator-5 kit (Zymo) according to the manufacturer’s instructions.

### RT–PCR with DMS-Modified RNA.

The methylated RNA was reverse transcribed as follows. First, 0.2 μM DMS-modified RNA, 2 μL 5 × first strand buffer (ThermoFisher Scientific), 1 μL 10 μM reverse primer (miR_tail_RT, *SI Appendix*, Table S8), 1 μL dNTP, 0.5 μL 0.1 M DTT, 0.5 μL RNaseOUT, and 0.5 μL thermostable group II intron reverse transcriptase, 3^rd^ generation (TGIRT-III, Ingex) were mixed. The mixture was incubated at 57 °C for 30 min. After the 30 min incubation, the temperature was increased to 85 °C for 5 min. Then, 1 μL RNase H (New England Biolabs) was added to the mixture to digest the RNA. The reverse-transcribed DNA was PCR amplified using Phusion (NEB) for 27 cycles according to the manufacturer’s instruction using primers miR31_buffer_F and miR_tail_RT (*SI Appendix*, Table S8). The PCR product was purified by GeneJET PCR purification kit (ThermoFisher Scientific).

### DMS-MaPseq of pre-miR-31 RNA.

Illumina sequencing adapters were added by ligation-mediated PCR using the NEBNext UltraII DNA Library Prep Kit (New England BioLabs). The libraries were Bioanalyzed on a high sensitivity DNA chip, size selected, and sequenced on Illumina Miseq 600 cycles (300x300 paired end). The resulting sequencing reads were adapter trimmed using Trim Galore and aligned using bowtie2 (“bowtie2–local–no-unal–no-discordant–no-mixed–phred33 40 -L 12”). Each read was compared to its reference sequence to count how many mutations occurred at each nucleotide. All sequencing reads were combined together to calculate the average mutations per base and create a mutational profile.

### Isotopic Labeling of RNAs for NMR.

Isotopically labeled RNAs were produced as described above by replacing the rNTP mixture with rNTPs of appropriate isotope labeling. ^15^N/^13^C rNTPs were obtained from Cambridge Isotope Laboratories (CIL, Andover, MA). The partially- and per-deuterated rNTPs used for in vitro transcription were obtained from Cambridge Isotope Laboratories (CIL, Andover, MA) or generated in house, as described below. Protiation at the C8 position of perdeuterated rGTP and rATP was achieved by incubation with triethylamine (TEA, 5 equiv) in H_2_O (60 °C for 24 h and for five days, respectively). Deuteration of the C8 position of fully protiated GTP and ATP was achieved by analogous treatment with D_2_O (99.8% deuteration; CIL). TEA was subsequently removed by lyophilization.

### NMR Experiments.

Samples for NMR experiments of Top, TopA, pre-miR-31, and FL pre-miR-31 were prepared in 300–350 μL 100% D_2_O (99.8% deuteration; CIL) or 10% D_2_O/90% H_2_O, 50 mM K-phosphate buffer (pH 7.5), 1 mM MgCl_2_ of 300–600 μM RNA in Shigemi NMR sample tubes. NMR spectra were collected on 600 and 800 MHz Bruker AVANCE NEO spectrometers equipped with a 5-mm three channel inverse (TCI) cryogenic probe (University of Michigan BioNMR Core). NMR spectra of Top and TopA were recorded at 30 °C and of pre-miR-31 and FL pre-miR-31 at 37 °C. The isotopic labeling scheme of FL pre-miR-31 used in specific NMR experiment is indicated in the figure legends. NMR data were processed with NMRFx (64) and analyzed with NMRViewJ (65). ^1^H chemical shifts were referenced to water and ^13^C chemical shifts were indirectly referenced from the ^1^H chemical shift (66).

The signals of nonexchangeable protons of Top and TopA were assigned based on analysis of 2D ^1^H–^1^H NOESY (τ_m_ = 400 ms), 2D ^1^H–^1^H TOCSY (τ_m_ = 80 ms), and ^1^H–^13^C HMQC spectra. Additionally, the 2D NOESY spectrum (τ_m_ = 400 ms) was recorded for A^H^C^H^-labeled Top RNA (A and C fully protiated, G and U perdeuterated). Nonexchangeable ^1^H assignments of FL pre-miR-31 were obtained from 2D NOESY data (τ_m_ = 400 ms) recorded on fully protiated FL pre-miR-31 and A^2r^G^r^-, A^2r^G^r^U^r^-, A^H^C^H^-, and G^H^U^6r^-labeled FL pre-miR-31 (superscripts denote sites of protiation on a given nucleoside, all other sites deuterated). ^1^H–^1^H TOCSY and ^1^H–^13^C HSQC spectra of ^15^N/^13^C AG-labeled FL pre-miR-31 were analyzed to facilitate the assignment. The NMR samples for pH titration were prepared with 300 μM ^15^N AU-labeled FL pre-miR-31 in 10% D_2_O/90% H_2_O, 1 mM MgCl_2_, and 10 mM K-phosphate buffer with pH values 5.8, 6.2, 6.5, 7.0, 7.5, and 8.0.

A best-selective long-range HNN-COSY (40) was recorded to identify AU base pairing in FL pre-miR-31. The spectrum was recorded on 560 μM ^15^N AU-labeled FL pre-miR-31 in 10% D_2_O/90% H_2_O, 50 mM K-phosphate buffer (pH = 7.5) and 1 mM MgCl_2_. Then, 64 complex points were recorded with a sweep width of 7.4 kHz for ^15^N, and 2,048 complex points with a sweep width of 16.6 kHz for ^1^H, 1,368 scans per complex increment at 37 °C and 800 MHz.

NMR sPRE (67) data of FL pre-miR-31 were obtained by measuring R1 relaxation rates (68) as a function of the concentration of paramagnetic compound Gd(DTPA-BMA) (69). We acquired ^1^H–^13^C HSQC-based pseudo-3D experiments at 0.0, 0.8, 1.6, 2.4, 3.2, and 4.8 mM concentration of the paramagnetic compound. Data were acquired on sample containing 480 μM ^15^N/^13^C A, G-labeled FL pre-miR-31 in 100% D_2_O (99.8% deuteration; CIL), 50 mM K-phosphate buffer (pD = 7.5), and 1 mM MgCl_2_ at 800 MHz using nine delays (0.02-2 s) with two repetitions at every titration point. The data were processed and analyzed using NMRFx (64). The sPRE values were obtained from the peak intensities of well-resolved peaks in the ^1^H–^13^C HSQC-based pseudo-3D experiments. These intensities were fitted to an exponential function (Eq. **1**) (68)

[1] $$I=Ae^{-xR_{1}}$$

where *I* is the intensity of the peak, *A* is the amplitude of the relaxation, and *R*_1_ is the longitudinal proton relaxation rate. The sPRE values were obtained from the *R*_1_ rates determined in the presence of different concentrations of paramagnetic compound Gd(DTPA-BMA) (Eq. **2**) (67)

[2] $$R_{1}\left(c_{\mathrm{Gd}}\right)=m_{\mathrm{sPRE}}+R_{1}^{0}$$

where *R*_1_(*c*_Gd_) is the *R*_1_ measured at the concentration of the paramagnetic compound (*c*_Gd_), the slope ms_PRE_ corresponds to the sPRE, and $R_{1}^{0}$ is the fitted *R*_1_ in the absence of the paramagnetic compound. The error of the sPRE value Δ*m*_sPRE_ was obtained from the linear regression as described previously (67).

^1^H–^13^C RDCs were recorded using IPAP-HSQC experiments (70) for ^15^N/^13^C AG-labeled FL pre-miR-31. Two samples were prepared, an isotropic sample containing 400 μM RNA in 90% H_2_O/10% D_2_O, 50 mM K-phosphate buffer (pH = 7.5), and 1 mM MgCl_2_ and an anisotropic sample containing 600 μM FL pre-miR-31 in the same solvent but also including 10 mg/mL Pf1 phage, yielding a solvent ^2^H quadrupole splitting of 11 Hz. Then, 110 complex points were recorded with a sweep width of 8 kHz for ^13^C and 32,768 complex points with a sweep width of 14.7 kHz for ^1^H, 200 scans per complex increment at 800 MHz. Spectra were processed and analyzed with Bruker Topspin.

### Structure Calculations.

CYANA was used to generate 640 initial structures via simulated annealing molecular dynamics calculations over 128,000 steps. Upper limits for the NOE distance restraints generally set at 5.0 Å for weak, 3.3 Å for medium, and 2.7 Å for strong signals, based on peak intensity. Notable exceptions included intraresidue NOEs between H6/H8 and H2′ (4.0 Å) and H3′ (3.0 Å). For very weak signals, 6.0 Å upper limit restraints were used, including for sequential H1′–H1′ NOEs and intraresidue H5–H1′ NOEs. Standard torsion angle restraints were included for regions with A-helical geometry, allowing for ±25° deviations from ideality (ζ = −73°, α = −62°, β = 180°, γ = 48°, δ = 83°, ɛ = −152°). Torsion angles for mismatches were further relaxed to allow for ±75° deviation from ideality. Hydrogen bonding restraints were included for experimentally validated base pairs as were standard planarity restraints. Cross-strand P–P distance restraints were employed for A-form helical regions to prevent the generation of structures with collapsed major grooves (71). A grid search was performed over a broad range of tensor magnitude and rhombicity with weighting of the experimentally determined ^1^H–^13^C residual dipolar couplings (RDCs) constraints. Forty input structures were further minimized after singular value decomposition fits of the RDC weights.

The top 20 CYANA-derived structures were then subjected to molecular dynamics simulations and energy minimization with AMBER (72). Only upper limit NOE, hydrogen bond, and dipolar coupling restraints were used, along with restraints to enforce planarity of aromatic residues and standard atomic covalent geometries and chiralities (71, 73). Backbone torsion angle and phosphate-phosphate restraints were excluded during AMBER refinement. Calculations were performed using the RNA.OL3 (74) and generalized Born (75) force fields. NMR restraints and structure statistics are presented in *SI Appendix*, Table S2.

### Small-Angle X-Ray Scattering (SAXS).

SAXS with in-line size exclusion chromatography (SEC) and multiangle light scattering (MALS) was performed at BioCAT (beamline 18ID at the Advanced Photon Source, Chicago). SAXS buffer contained 50 mM potassium phosphate buffer, pH 7.5, 50 mM NaCl, 1 mM MgCl_2_, and all data were collected at 20 °C. Full details of SAXS data collection and analysis are presented in *SI Appendix* and *SI Appendix*, Table S3.

### ^32^P Labeling of RNA.

The 5′-end labeling of RNA was performed using 5 pmol of RNA, 1 μL γ-^32^P-ATP (PerkinElmer), and 10 U T4 polynucleotide kinase (New England Biolabs) in a final volume of 10 µL. Before labeling, RNA was boiled for 3 min and snap-cooled by placing on ice for another 3 min. The radiolabeled RNA was purified on a G-25 column (Cytiva) according to the manufacturer’s instructions. The radiolabeled RNA concentration was determined based on a standard curve which was obtained from the counts per minute of the γ-^32^P-ATP source.

### Dicer and Dicer–TRBP Processing Assay.

Human Dicer protein processing assay was performed as previously described with minimal modifications (20). Concentrated recombinant human Dicer protein was diluted in 1X Dicing buffer (24 mM HEPES or 24 mM Bis-Tris, pH 7.5, 100 mM NaCl, 5 mM MgCl_2_, 4 μM EDTA). Dicer enzyme was premixed with 80 U RNaseOUT Recombinant Ribonuclease Inhibitor (Thermo Fisher Scientific) and 5X dicing buffer (120 mM HEPES or 120 mM Bis-Tris, pH 7.5, 0.5 M NaCl, 25 mM MgCl_2_, 0.02 mM EDTA). The ^32^P-labeled RNA was heated to 95 °C for 3 min and then placed on ice for another 3 min. The RNA (1 μL) was added to premixed solution (9 μL) and incubated at 37 °C. The final RNA and enzyme concentration are 2 nM and 20 nM, respectively. The reaction is quenched by adding 10 μL quench buffer (98% Formamide, 20 mM EDTA, trace bromophenol blue and xylene cyanol) at 20, 40, 60, 120, 180, 240, 300, 420, and 600 s, respectively. After sample was run on a 12% denaturing polyacrylamide gel, the gel was exposed to a phosphor screen, which was scanned by a Typhoon Phosphor Imager (GE Healthcare). The gel image was quantified analyzed by ImageJ. The Dicer cleavage ratio was calculated as the sum of the intensity of products and partially digested products divided by the sum of the intensity of the products, partially digested products, and remaining substrate. Experiments were performed in triplicate. The average and SD of the measurements are reported. Processing assays with the reconstituted Dicer–TRBP complex were conducted as described for Dicer alone.

The pre-miRNA cleavage ratio was fitted as function of time to get apparent rate constant (*k*_obs_) using Graphpad Prism Software. Data are fitted using Eq. **3**:

[3] $$Cleavage\;ratio=Y_{0}+A\times \left(1-e^{-k_{\mathrm{obs}}t}\right)$$

In the fitting parameter, *Y*_0_ and *A* are global nonregressive fitting and shared among data set, *Y*_0_ is set to be less than 5, and *A* is set to be less than 100. A Student’s *t* test was used to compare the means of the calculated *k*_obs_ between mutant and WT RNAs.

### CD-Thermal Denaturation of RNA and Data Analysis.

CD-thermal denaturing of RNAs were performed on a JASCO J1500CD spectrometer with a heating rate of 1 °C per min from the 5 °C to 95 °C. Data points were collected every 0.5 °C with absorbance detection at 260 nm. Then, 20 μM RNA samples were premixed in potassium phosphate buffer (pH = 7.5) with 1 mM MgCl_2_. The single transition unfolding melting profiles were analyzed using a two-state model using sloping baselines (Eq. **4**) (76).

[4] $$f\left(T\right)=\frac{\left(m_{u}T+b_{u}\right)+{\left(m_{f}T+b_{f}\right)e}^{\left[\frac{\Delta H}{R}\right]\left[\frac{1}{\left(T_{m}+273.15\right)}-\frac{1}{\left(T+273.15\right)}\right]}}{1+e^{\left[\frac{\Delta H}{R}\right]\left[\frac{1}{\left(T_{m}+273.15\right)}-\frac{1}{\left(T+273.15\right)}\right]}}$$

where m_u_ and m_f_ are the slopes of the lower (unfolded) and upper (folded) baselines and *b*_u_ and *b*_f_ are the y-intercepts of the lower and upper baselines, respectively. Δ*H* (in kcal/mol) is the enthalpy of folding, *T*_m_ (in °C) is the melting temperature, and R is the gas constant (0.001987 kcal/(Kmol)). Experiments were performed in triplicate. The average and SD of the measurements are reported.

## Supplementary Material

Appendix 01 (PDF)

## Data Availability

Resonance assignments have been deposited in the BMRB (miR-31_TopA: 51697 (77), miR-31_Top: 51698 (78), pre-miR-31: 31061 (79)). NMR-derived structures have been deposited in the PDB (pre-miR-31: 8FCS (80)). Experimental SAXS data of pre-miR-31 have been deposited in the Small Angle Scattering Biological Data Bank under accession code SASDRF9 (81). Raw sequencing data were deposited in BioSample (31573939) (82).
