# Supplementary material for: Structure of pre-miR-31 reveals an active role in Dicer–TRBP complex processing
Source: Proc Natl Acad Sci U S A. 2023 Sep 19;120(39):e2300527120. doi: 10.1073/pnas.2300527120 (PMC10523476; doi:10.1073/pnas.2300527120)
Supplement: Supplementary file 1 — Appendix 01 (PDF) [file pnas.2300527120.sapp.pdf]

PNAS

Supporting Information for
Structure of pre-miR-31 reveals an active role in Dicer-TRBP complex processing.

Sicong Ma[1#], Anita Kotar[1,2#], Ian Hall[3], Scott Grote[4], Silvi Rouskin[4] and Sarah C. Keane[1,3*]

[1]Biophysics Program, University of Michigan, 930 N. University Avenue, Ann Arbor, MI 48109, USA

[2]Current Address: Slovenian NMR Centre, National Institute of Chemistry, Hajdrihova 19, SI-1000 Ljubljana, Slovenia

[3]Department of Chemistry, University of Michigan, 930 N. University Avenue, Ann Arbor, MI 48109, USA

[4]Department of Microbiology, Harvard Medical School, Boston, MA 02115, USA

[#]Authors contributed equally

Sarah C. Keane
Email: sckeane@umich.edu

This PDF file includes:

- Supporting text
- Figures S1 to S24
- Tables S1 to S13
- SI References

Supporting Information Text

Expanded Methods for Small angle X-ray scattering (SAXS). Pre-miR-31 RNA (225 µL, 2.31 mg/mL) was loaded on a Superdex 75 Increase 10/300 GL column (Cytiva) run by a 1260 Infinity II HPLC (Agilent Technologies) at 0.6 mL/min. The eluate passed sequentially through the Agilent UV detector, a MALS detector and a dynamic light scattering (DLS) detector (DAWN Helios II, Wyatt Technologies), and a refractive index (RI) RI detector (Optilab TrEX, Wyatt). The eluate then flowed through the SAXS flow cell consisting of a 1.0 mm ID quartz capillary with ≈ 20 µm walls. A coflowing buffer sheath is used to separate sample from the capillary walls to help prevent radiation damage (1). Scattering intensity was recorded using an Eiger2 XE 9M detector (Dectris) which was placed 3.688 m from the sample giving access to a q-range of 0.0027 to 0.42 $Å^{-1}$. 0.2 s exposures were collected every 1 s during elution and data was reduced using BioXTAS RAW 2.1.4 (2). The buffer blank was created by averaging frames preceding the elution peak and subtracted from exposures selected from the elution peak to create the I(q) vs q curves used for subsequent analysis. Molecular weights and hydrodynamic radii were calculated from the MALS and DLS data respectively using the ASTRA 7 software (Wyatt). The GNOM package (3) was used to determine the pair-distance distribution function [P(r)] required for molecular reconstruction. The maximum linear dimension of the molecule, D_{max}, is calibrated for goodness-of-fit by enforcing a smooth zeroing of $P(D_{max})$. DENNS was used to calculate the *ab initio* electron density map directly from the GNOM output. 20 reconstructions were performed in slow mode with default parameters and averaged. Alignment of the reconstructions to the structure was done with the DENNS alignment tool in BioXTAS Raw. Reconstructions were visualized using Pymol 2.5.5. FoXS (4, 5) was used to back calculate the scattering profile of pre-miR-31 and to compare the back-calculated scattering to the experimental scattering.

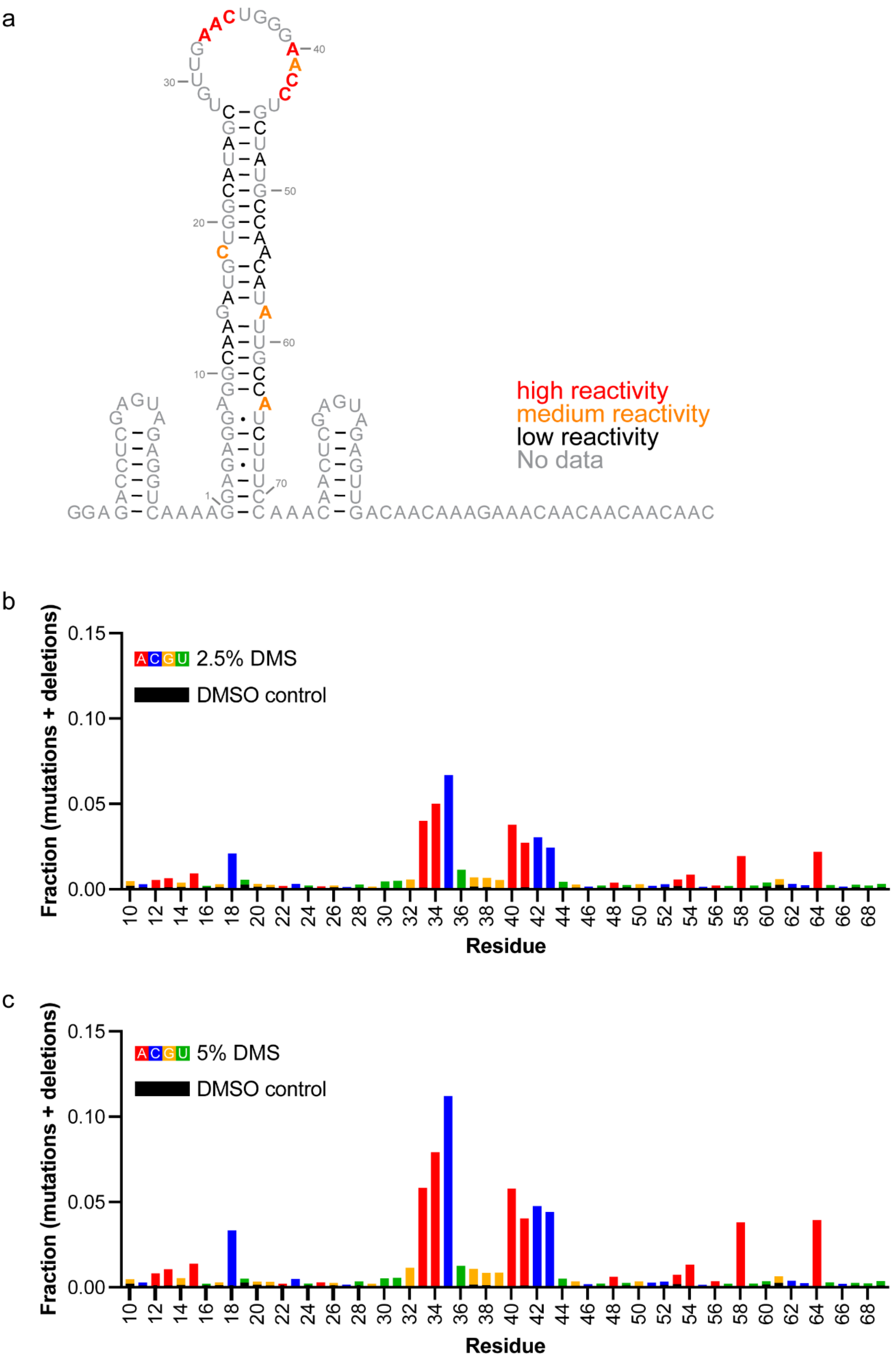
a
high reactivity
medium reactivity
low reactivity
No data
b
ACGU 2.5% DMS
DMSO control
Fraction (mutations + deletions)
Residue
c
ACGU 5% DMS
DMSO control
Fraction (mutations + deletions)
Residue

Fig. S1. *In vitro* DMS-MaPseq of pre-miRNA-31 RNA. a) Construct design and reactivity scores. 5' and 3' extensions were included to facilitate library preparation and sequencing. These regions were not predicted to disrupt the folding of pre-miR-31. b and c) Fraction of mutations and deletions on a per-residue basis upon reaction with 2.5% DMS (b) or 5% DMS (c). A control where DMSO was included in the reaction rather than DMS indicates minimal background mutations (black bars in b and c). Secondary structure was rendered using RNA2Drawer (6)

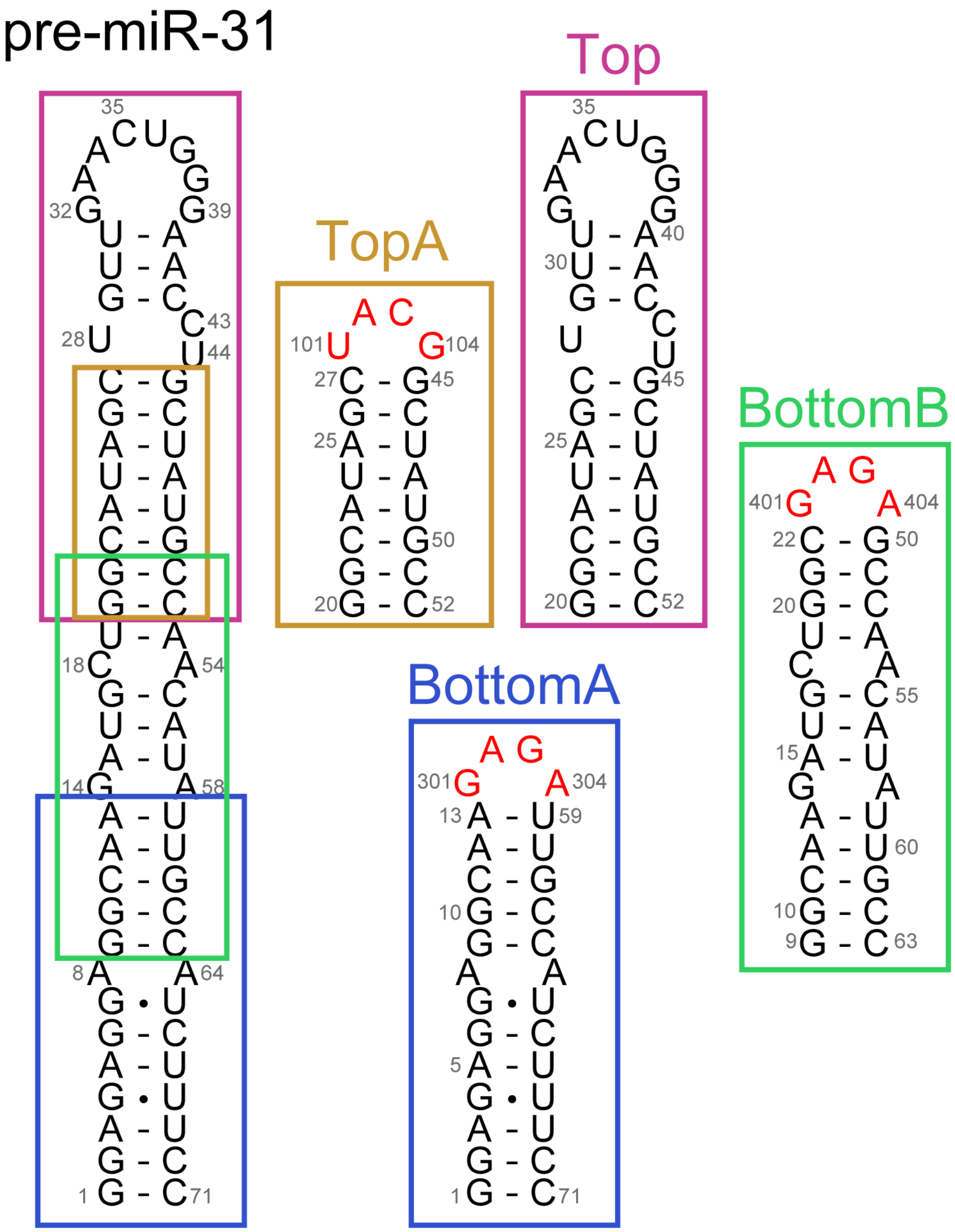

Fig. S2. Oligo controls for NMR chemical shift assignment of FL-pre-miR-31. Four oligonucleotide controls were designed to cover the entire FL-pre-miR-31 sequence. Non-native tetraloops (red) were included to cap oligos that truncated the apical loop. Secondary structures were rendered using RNA2Drawer (6).

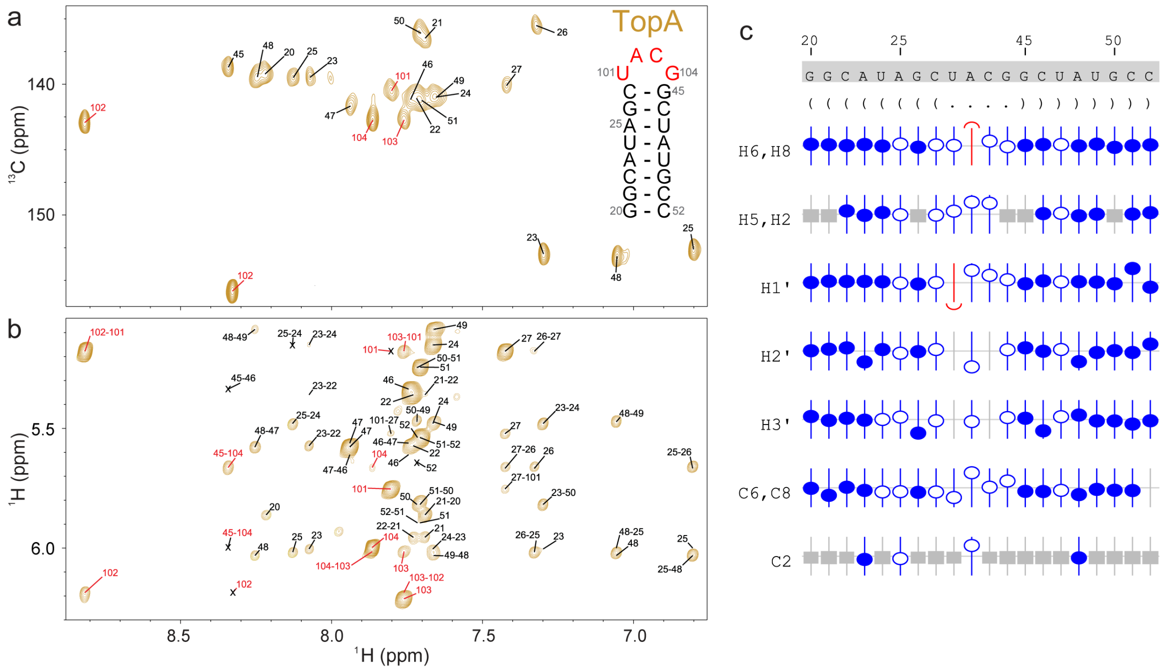

Fig. S3. Assigned chemical shifts of TopA RNA. a) ^{1}H-^{13}C HMQC and **b)** ^{1}H-^{1}H NOESY of TopA RNA. The signals assigned to GAGA tetraloop are colored red. NMR spectra were recorded at 0.4 mM RNA concentration, 50 mM K-phosphate buffer, pH=7.5, 1 mM $MgCl_2$ and 100% D_2O. **c)** Sequence analysis and validation of TopA chemical shift assignments. Nucleotide numbering, sequence, and secondary structure in Vienna format for TopA RNA. NMRViewJ chemical shift prediction software was used to validate proton (H6/H8, H5/H2, H1´, H2´, H3´) and carbon (C6/C8, C2) assignments. Assigned atoms are represented with blue circles (open and closed), while grey boxes denote atoms that are not present in a given base. Deviation from the predicted chemical shift is shown with the offset from the center. Filled circles indicate that there are chemical shifts for atoms with the same set of attributes in the BMRB. Open circles indicate atoms that have a prediction, but for which no exact matches of the attributes are available in the BMRB. Secondary structures were rendered using RNA2Drawer(6).

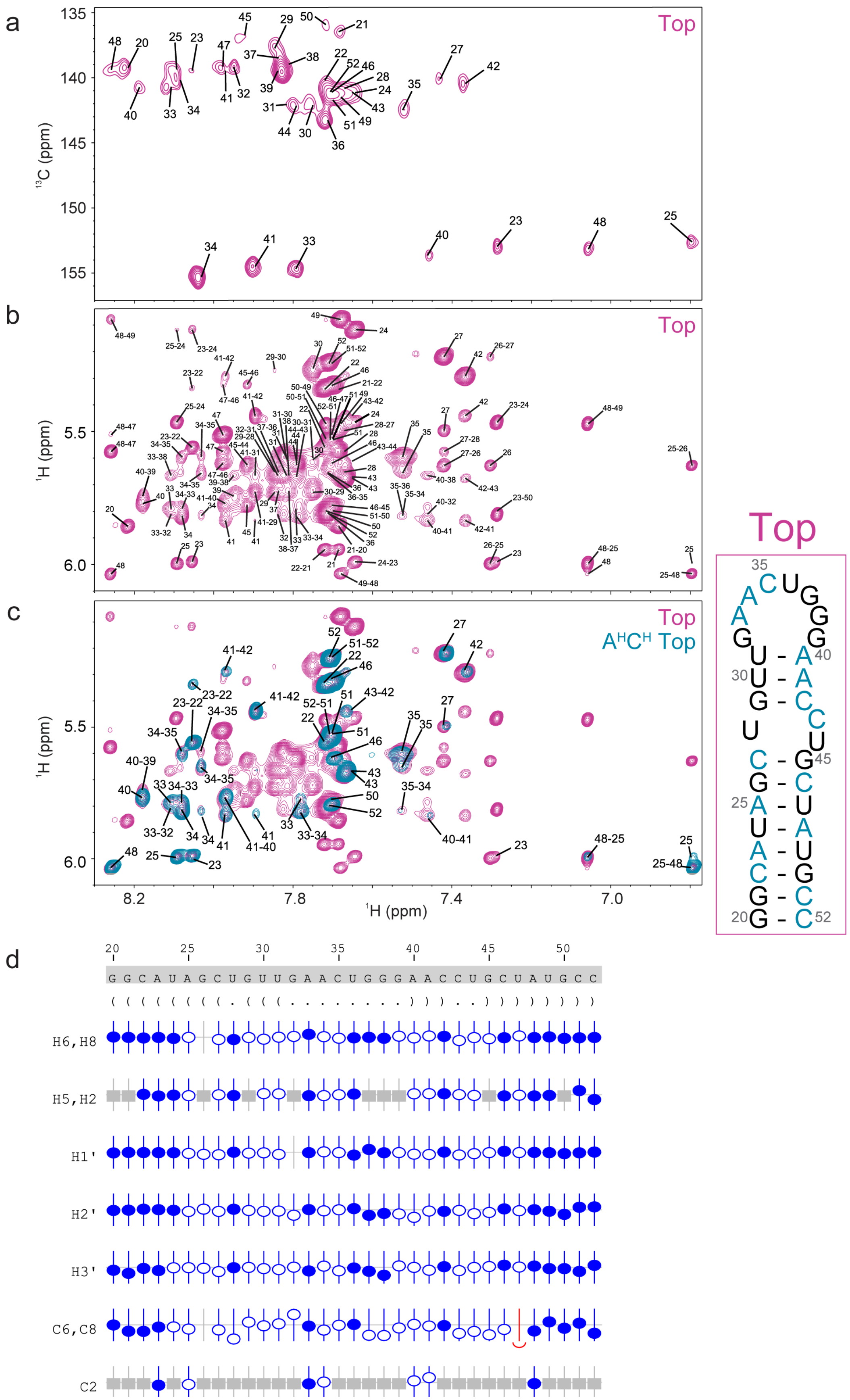

a
b
c
d
Top
AHCH Top
13C (ppm)
1H (ppm)
GGCAUAGCUGUUGAACUGGGAACCUGCUAUGCC
H6,H8
H5,H2
H1'
H2'
H3'
C6,C8
C2

Fig. S4. Assigned chemical shifts of Top RNA. a) ^{1}H-^{13}C HMQC and **b)** ^{1}H-^{1}H NOESY of Top RNA. **c)** ^{1}H-^{1}H NOESY spectrum overlay of fully-protiated (pink) and $A^{H}C^{H}$-labeled (teal) Top RNA. Secondary structure is colored to indicate the proton position (teal) in the $A^{H}C^{H}$-labeled Top RNA sample. All other sites are perdeuterated (black). NMR spectra were recorded at 0.4 mM RNA concentration, 50 mM K-phosphate buffer, pH=7.5, 1 mM $MgCl_2$ and 100% D_2O. **d)** Sequence analysis and validation of Top chemical shift assignments. Nucleotide numbering, sequence, and secondary structure in Vienna format for Top RNA. NMRViewJ chemical shift prediction software was used to validate proton (H6/H8, H5/H2, H1´, H2´, H3´) and carbon (C6/C8, C2) assignments. Assigned atoms are represented with blue circles (open and closed), while grey boxes denote atoms that are not present in a given base. Deviation from the predicted chemical shift is shown with the offset from the center. Filled circles indicate that there are chemical shifts for atoms with the same set of attributes in the BMRB. Open circles indicate atoms that have a prediction, but for which no exact matches of the attributes are available in the BMRB.

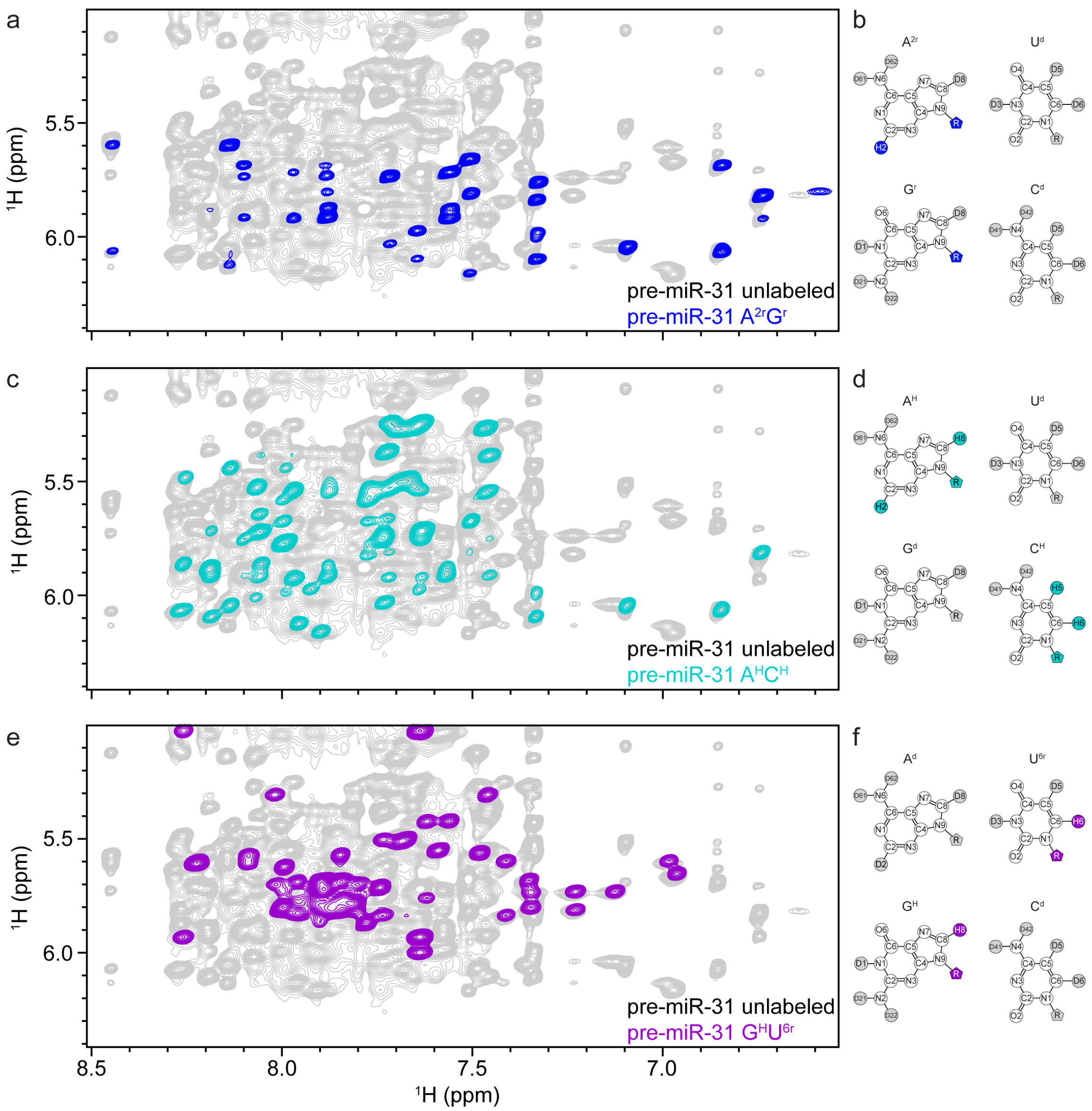

Fig. S5. Deuterium labeling improves spectral quality by reducing overlap. a) Overlay of the unlabeled (fully protiated, gray) and $A^{2r}G^{r}$-labeled (blue) pre-miR-31 ^{1}H-^{1}H NOESY spectra. **b)** Chemical structures of the four nucleosides indicating sites of selective deuteration (gray shade). Sites containing non-exchangeable protons are colored blue. **c)** Overlay of the unlabeled (fully protiated, gray) and $A^{H}C^{H}$-labeled (teal) pre-miR-31 ^{1}H-^{1}H NOESY spectra. **d)** Chemical structures of the four nucleosides indicating sites of selective deuteration (gray shade). Sites containing non-exchangeable protons are colored teal. **e)** Overlay of the unlabeled (fully protiated, gray) and $G^{H}U^{6r}$-labeled (purple) pre-miR-31 ^{1}H-^{1}H NOESY spectra. **f)** Chemical structures of the four nucleosides indicating sites of selective deuteration (gray shade). Sites containing non-exchangeable protons are colored purple.

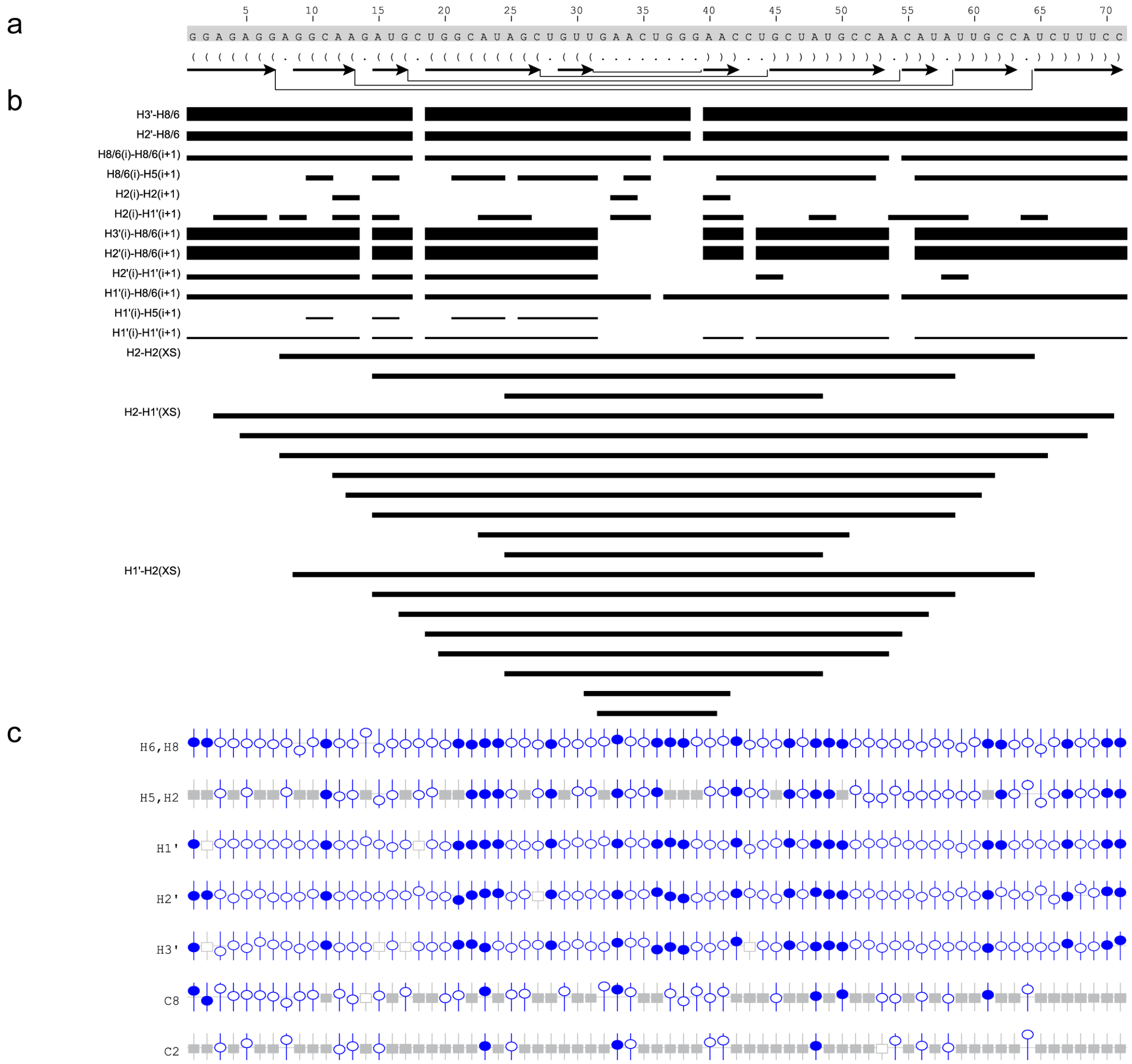

Fig. S6. Summary of the secondary structure, NOE connectivity, and chemical shift assignment validation for FL pre-miR-31. a) The secondary structure is shown beneath the sequence in Vienna format along with arrows to denote helical regions. **b)** NOE upper limit restraints for specified proton pairs used in CYANA and AMBER calculations are drawn as black bars. The thickness of the bar is representative of the strength of the measured NOE. **c)** NMRViewJ chemical shift prediction software was used to validate assignments of H6/H8, H5/H2, H1', H2', H3', C8 and C2. Protons that have been assigned in FL pre-miR-31 are indicated with blue circles (open and closed). Deviation from the predicted chemical shift is represented by deviation from the center. Predictions that are based on examples in the database of chemical shifts are shown as filled circles, predictions without data are shown as open circles. Filled grey squares are present in for nucleotides that do not contain a given proton or carbon. Unassigned resonances have open grey symbols.

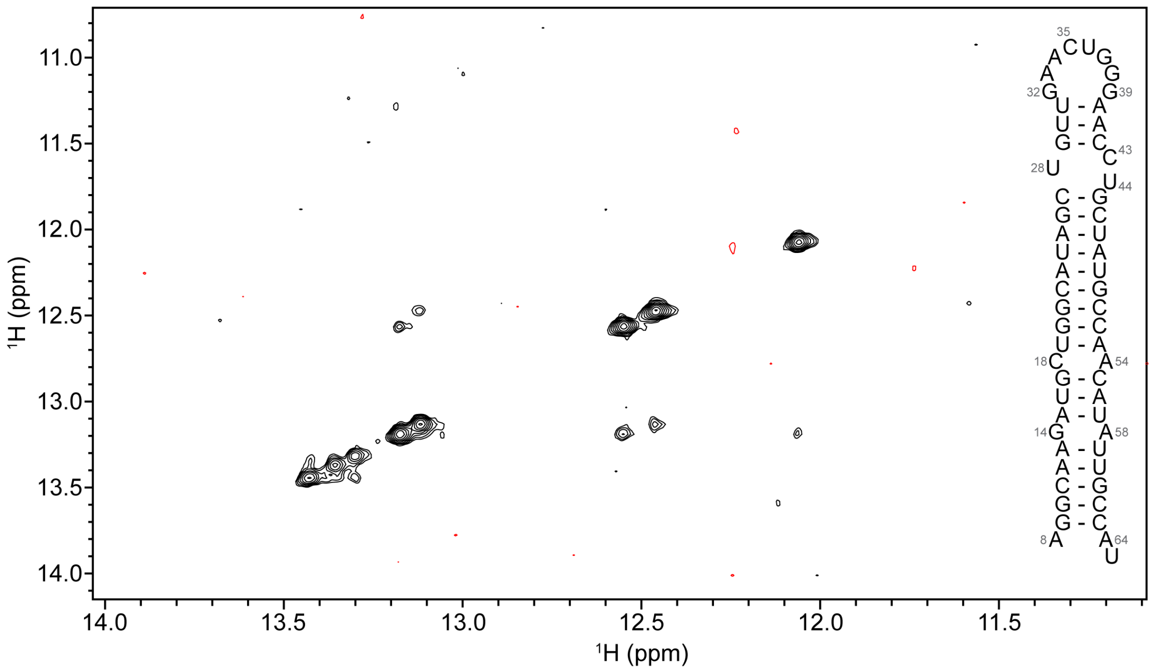

Fig. S7. Imino proton NOESY spectrum of pre-miR-31. The imino proton NOESY spectrum (mixing time 300 ms) of pre-miR-31 exhibited severe line broadening. The NMR spectrum was recorded at 0.33 mM RNA concentration, 50 mM K-phosphate buffer, pH=7.5, 1 mM $MgCl_2$ and 90% H_2O/10% D_2O at 37°C and 600 MHz.

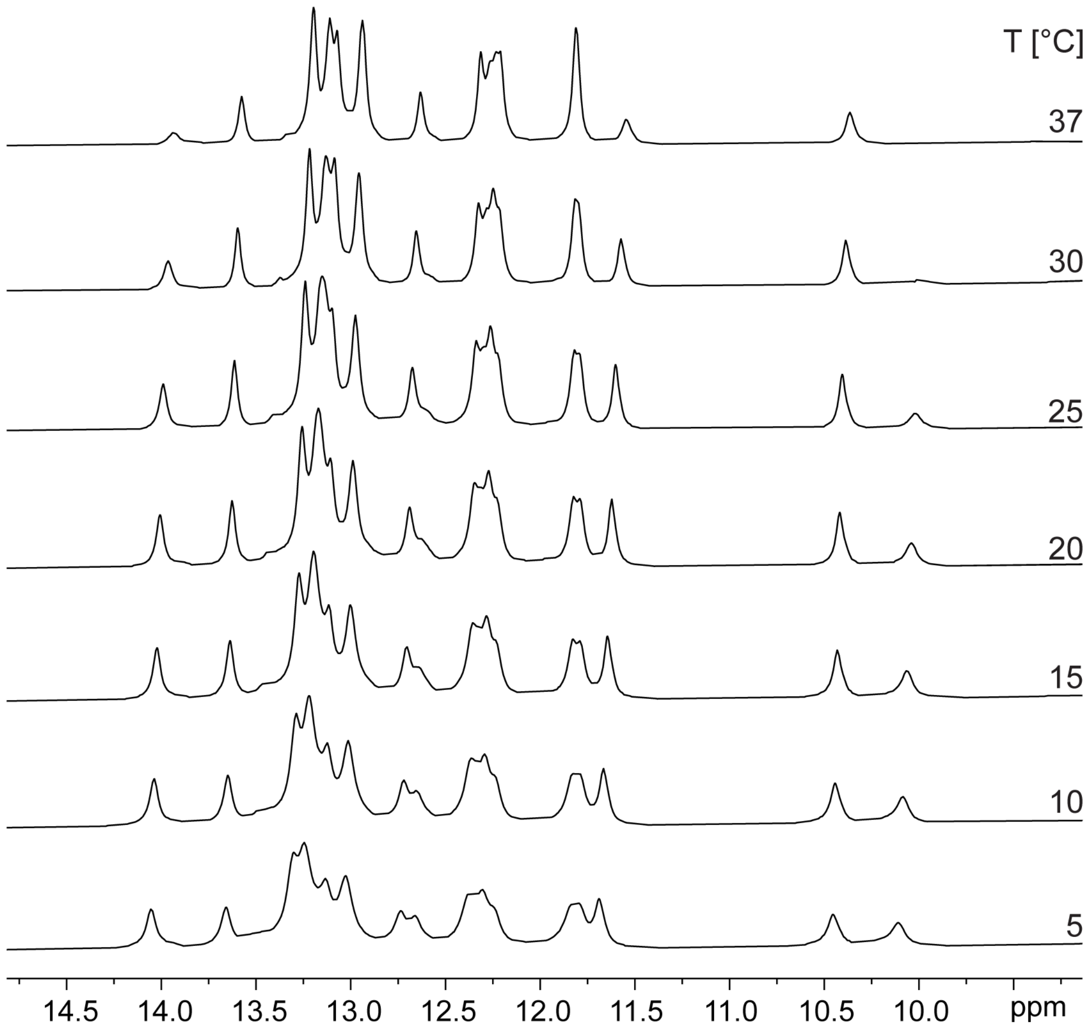

Fig. S8. Imino region of ^{1}H spectra of pre-miR-31 FL at different temperatures. The NMR spectra were recorded at 0.3 mM RNA concentration, 50 mM K-phosphate buffer, pH=7.5, 1 mM $MgCl_2$ and 90% H_2O/10% D_2O at 600 MHz and at temperatures between 5 and 37 °C as indicated on the right side of spectra.

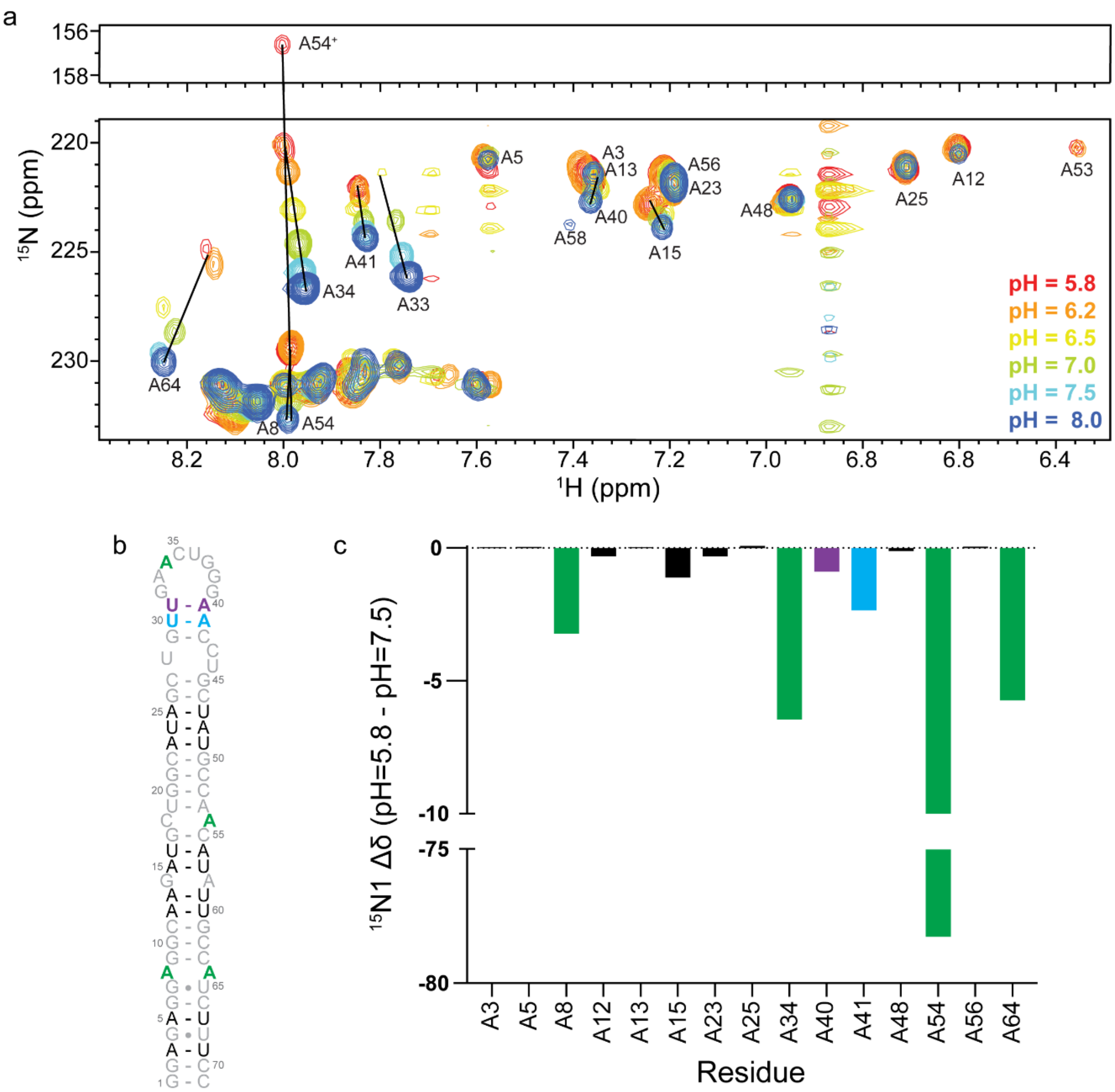

Fig. S9. pH-dependence of unpaired adenosines. a) BEST selective ^{1}H-^{15}N HSQC spectra of ^{15}N-AU labeled FL pre-miR-31, collected at various pH conditions. **b)** Secondary structure of FL pre-miR-31. **c)** Quantification of chemical shift perturbations (pH=5.8 – pH=7.5). A33 and A58 were not included in this analysis. The cross-peak of A33 is too broad to detect at pH=5.8 and cross-peak of A58 is severely overlapped at pH=5.8. Coloring follows the secondary structure in panel b.

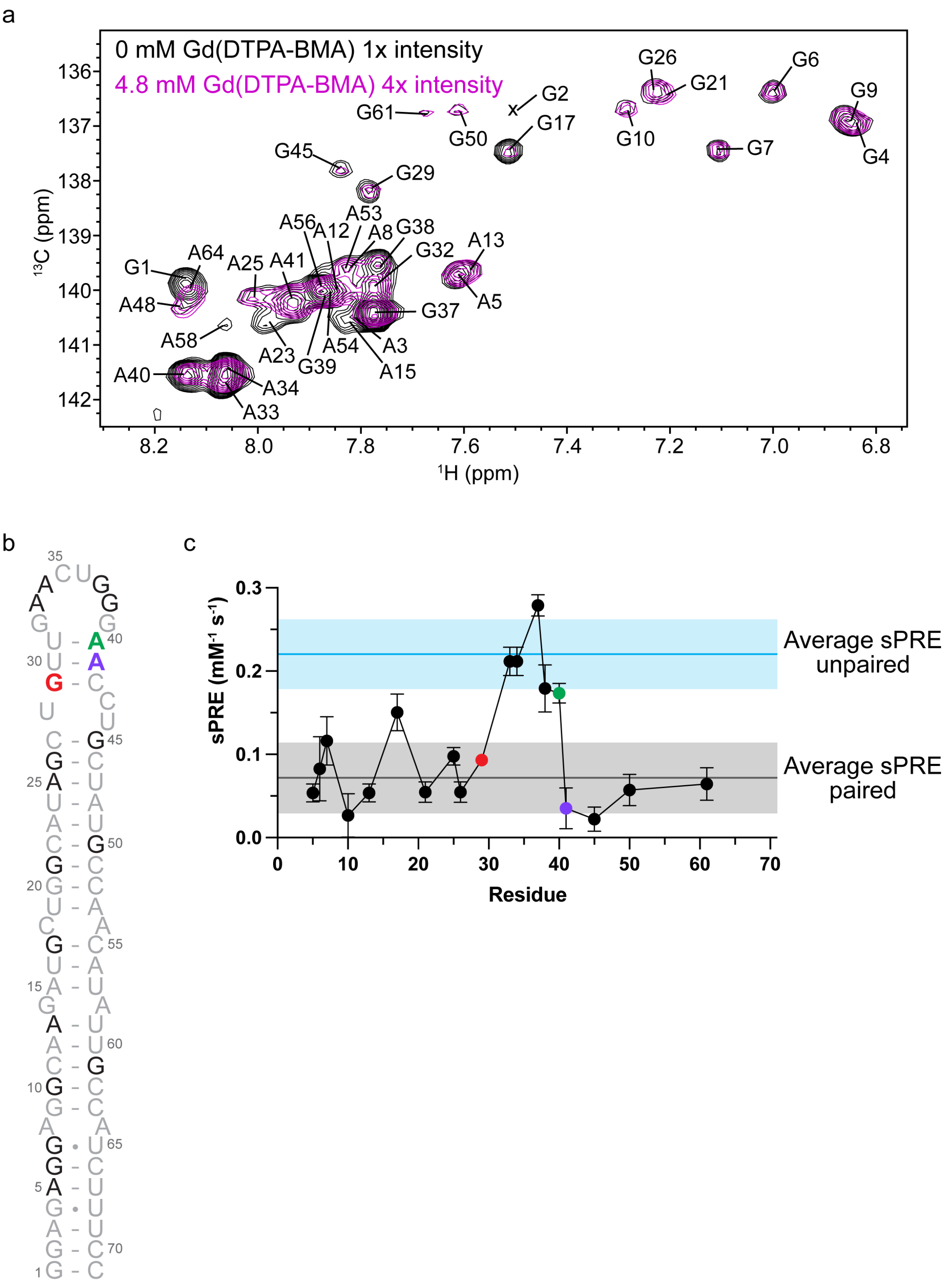

Fig. S10. Solvent paramagnetic relaxation effect analysis of FL pre-miR-31 reveals solvent accessibility in the loop region. a) ^{1}H-^{13}C HSQC spectra of ^{15}N/^{13}C A,G-labeled FL pre-miR-31 in the absence (black) and in the presence (magenta) of 4.8 mM paramagnetic compound Gd(DTPA-BMA). NMR spectra were recorded at 0.48 mM RNA concentration, 50 mM K-

phosphate buffer (pD=7.5), 1 mM $MgCl_2$ and 100% D_2O at 37 °C and 800 MHz. The H8-C8 assignments are labeled on the spectra. **b)** FL pre-miR-31 secondary structure colored to indicate the position of junction residues G29, A40 and A41 (red, green, and purple, respectively). Residues shaded gray were not included in the analysis. **c)** sPRE data for aromatic H8 protons of FL pre-miR-31. The errors of the sPRE values were obtained from the linear regression as described previously (7). The average sPRE values ± one standard deviation for unpaired (blue shading) and paired (grey shading) residues are indicated.

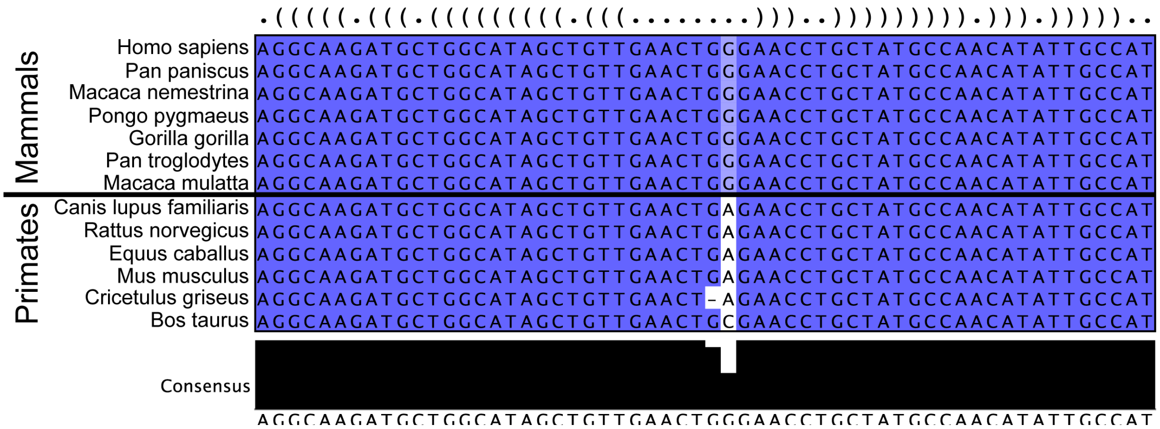

Fig. S11. Sequence conservation of pre-miR-31.

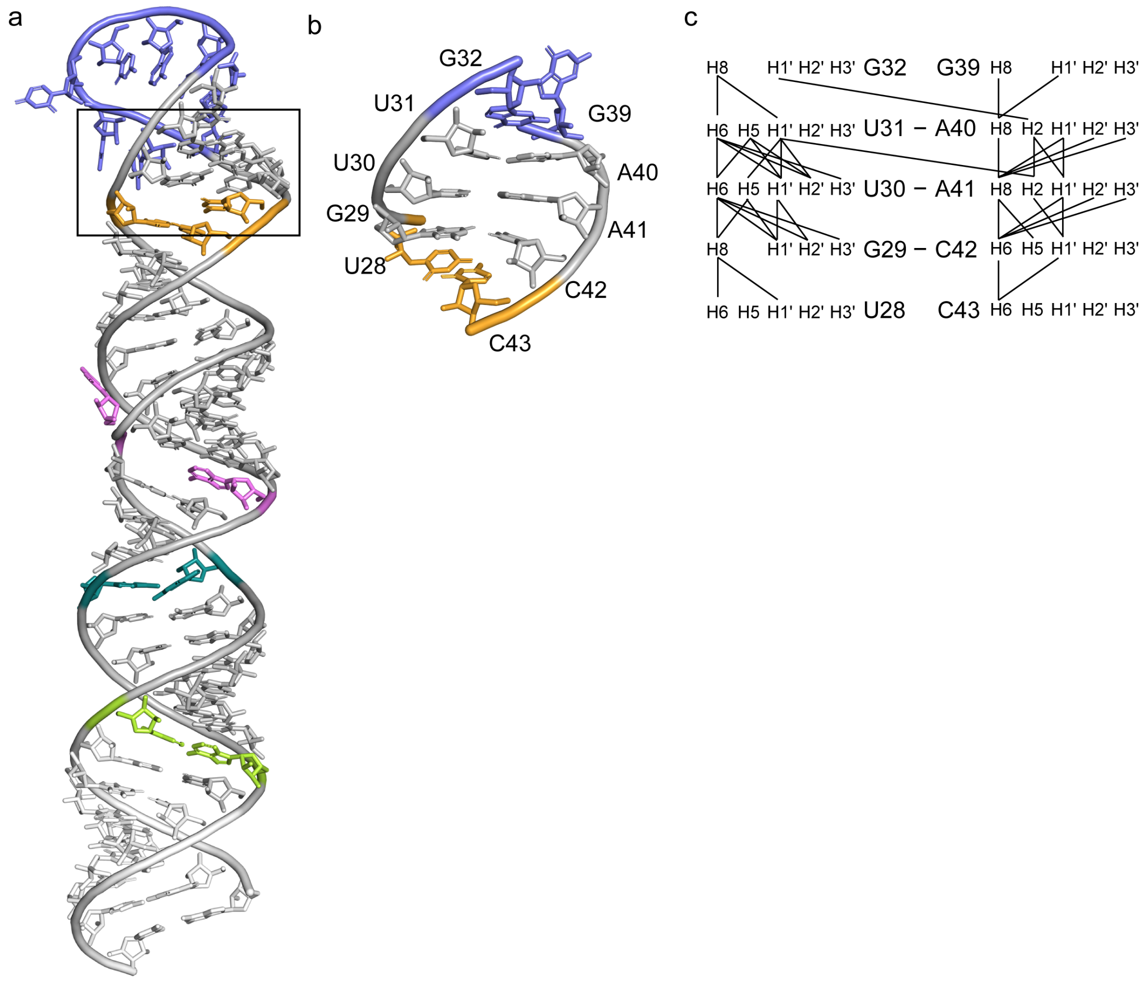

Fig. S12. Schematic presentation of inter-residue NOEs defining the pre-miR-31 junction base pairs. a) Structure of pre-miR-31 with junction region boxed. **b)** Expanded view of the junction. **c)** Schematic of residues, relevant atoms, and identified inter-residue NOEs used in structure calculations. The black lines represent the inter-residue NOEs.

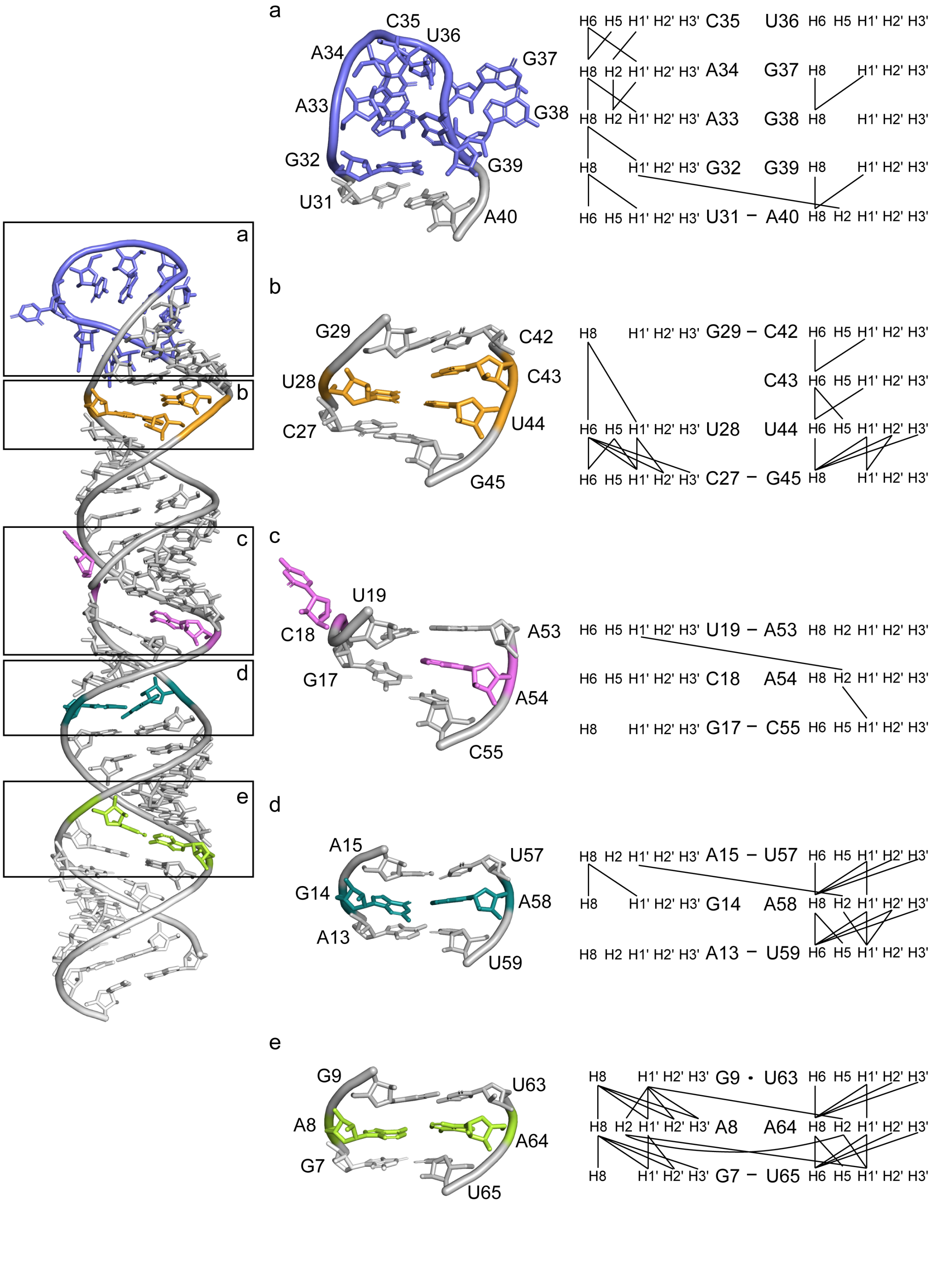

Fig. S13. Schematic presentation of inter-residue NOEs defines the apical and internal loops in pre-miR-31. (left) Structure of pre-miR-31 with mismatch/loop regions indicated.

(middle) Expanded view of the **a)** apical loop, **b)** dicing site, **c)** C•A mismatch, **d)** A•G mismatch, and **e)** A•A mismatch. **(right)** Schematic of residues, relevant atoms, and identified inter-residue NOEs used in structure calculations. The black lines represent the inter-residue NOEs.

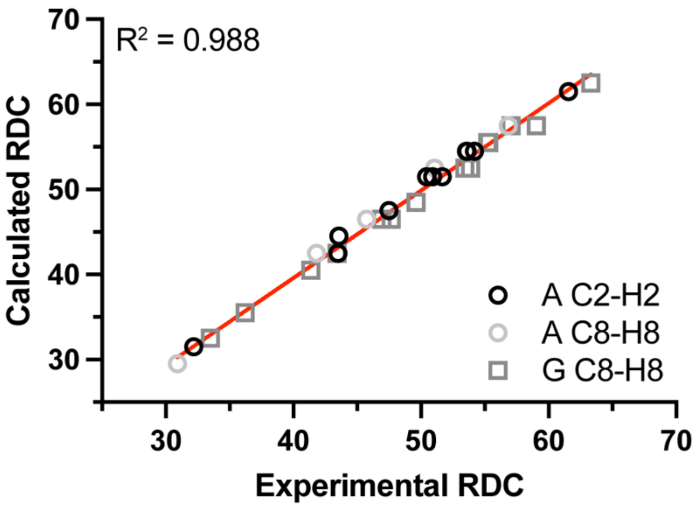

Fig. S14. Correlation plot between measured and back-calculated RDCs for the lowest energy pre-miR-31 structure.

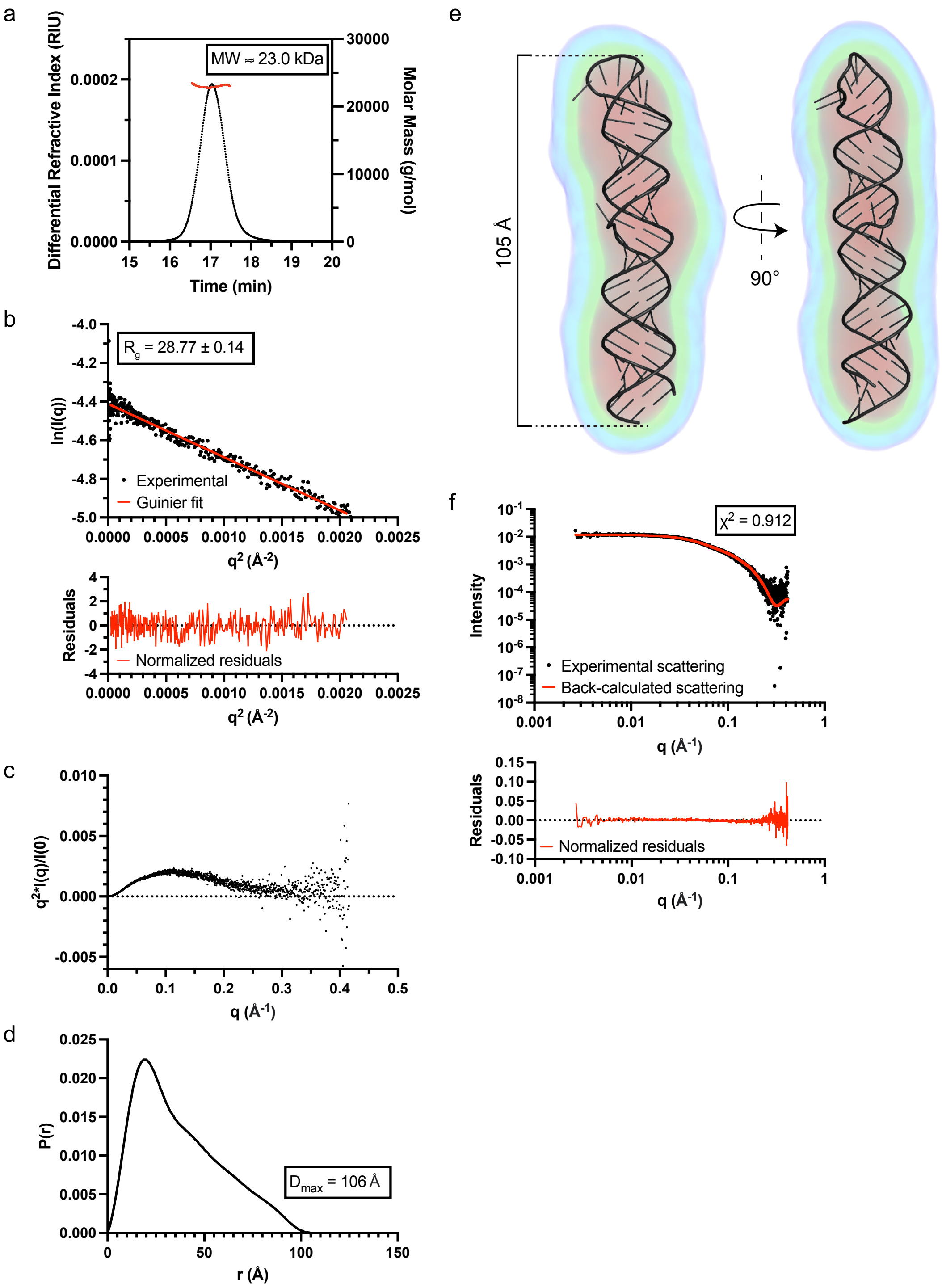

Fig. S15. Validation of pre-miR_31 tertiary structure by SAXS. a) SEC-MALS of pre-miR-31 FL. **b)** Guinier analysis of pre-miR-31 FL used to derive R_g and $I(0)$ parameters (top). Normalized residuals are flat and randomly distributed about zero (bottom). **c)** Dimensionless Kratky plot of pre-miR-31 FL. **d)** Pair distance distribution [P(r)] plot of pre-miR-31 FL. **e)** The lowest energy AMBER-refined pre-miR-31 FL structure aligned to the SAXS electron density map reconstruction. **f)** FoXS back-calculated scattering curve (red) of lowest energy structure fit to experimental SAXS data (black circles) (top). Normalized residuals are flat and randomly distributed about zero (bottom).

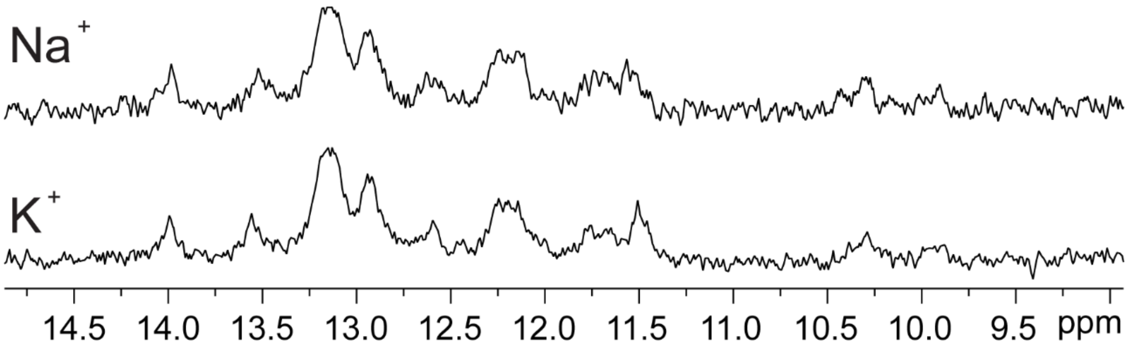

Fig. S16. Structure of pre-miR-31 is not sensitive to the monovalent cation. The NMR spectra were recorded at 0.01 mM RNA concentration, Na^+: 24 mM Na-phosphate buffer, pH=7.5, 100 mM NaCl, 5 mM $MgCl_2$, 4 μM EDTA; and K^+: 24 mM K-phosphate buffer, pH=7.5, 100 mM KCl, 5 mM $MgCl_2$, 4 μM EDTA, both at 10 °C and at 600 MHz.

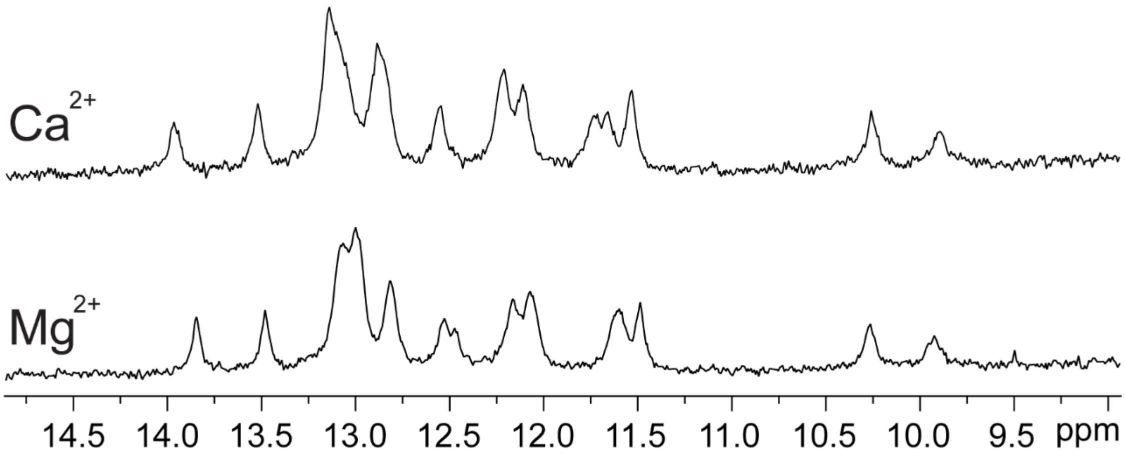

Fig. S17. **Structure of pre-miR-31 is not sensitive to the divalent cation.** The NMR spectra were recorded at 0.01 mM RNA concentration, in 24 mM K-phosphate buffer, pH=7.5, 5 mM $MgCl_2$ or 5 mM $CaCl_2$ (as indicated on the left) and 90% H_2O/10% D_2O at 10°C and at 600 MHz.

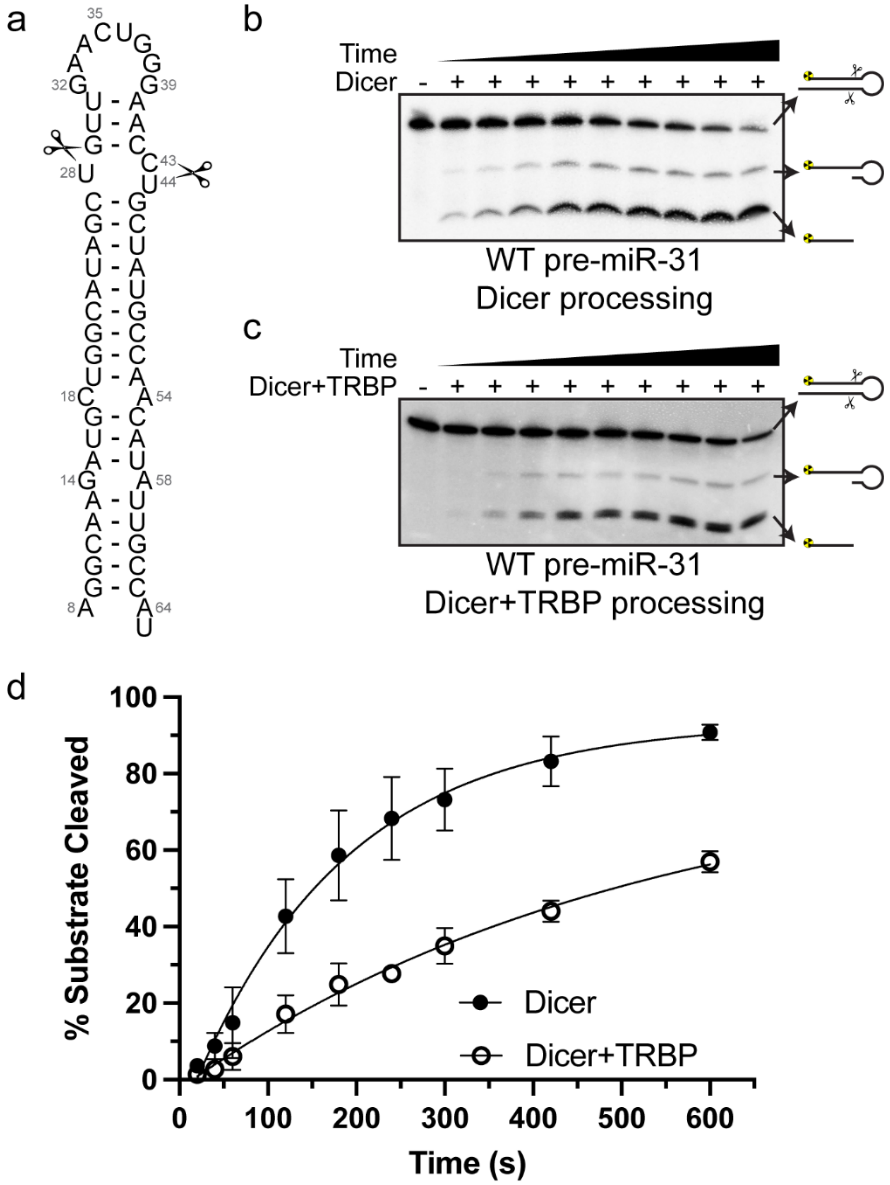

Fig. S18. Dicer processing co-factor, TRBP, inhibits pre-miR-31 processing. a) Secondary structures of pre-miR-31. **b)** Denaturing polyacrylamide gel resolving the RNA products upon incubation with Dicer. **c)** Denaturing polyacrylamide gel resolving the RNA products upon incubation with Dicer-TRBP complex. **d)** Quantification of pre-miR-31 processing with either Dicer (closed circles) or the Dicer-TRBP complex (open circles). Average and standard deviation from n=3 independent assays are presented.

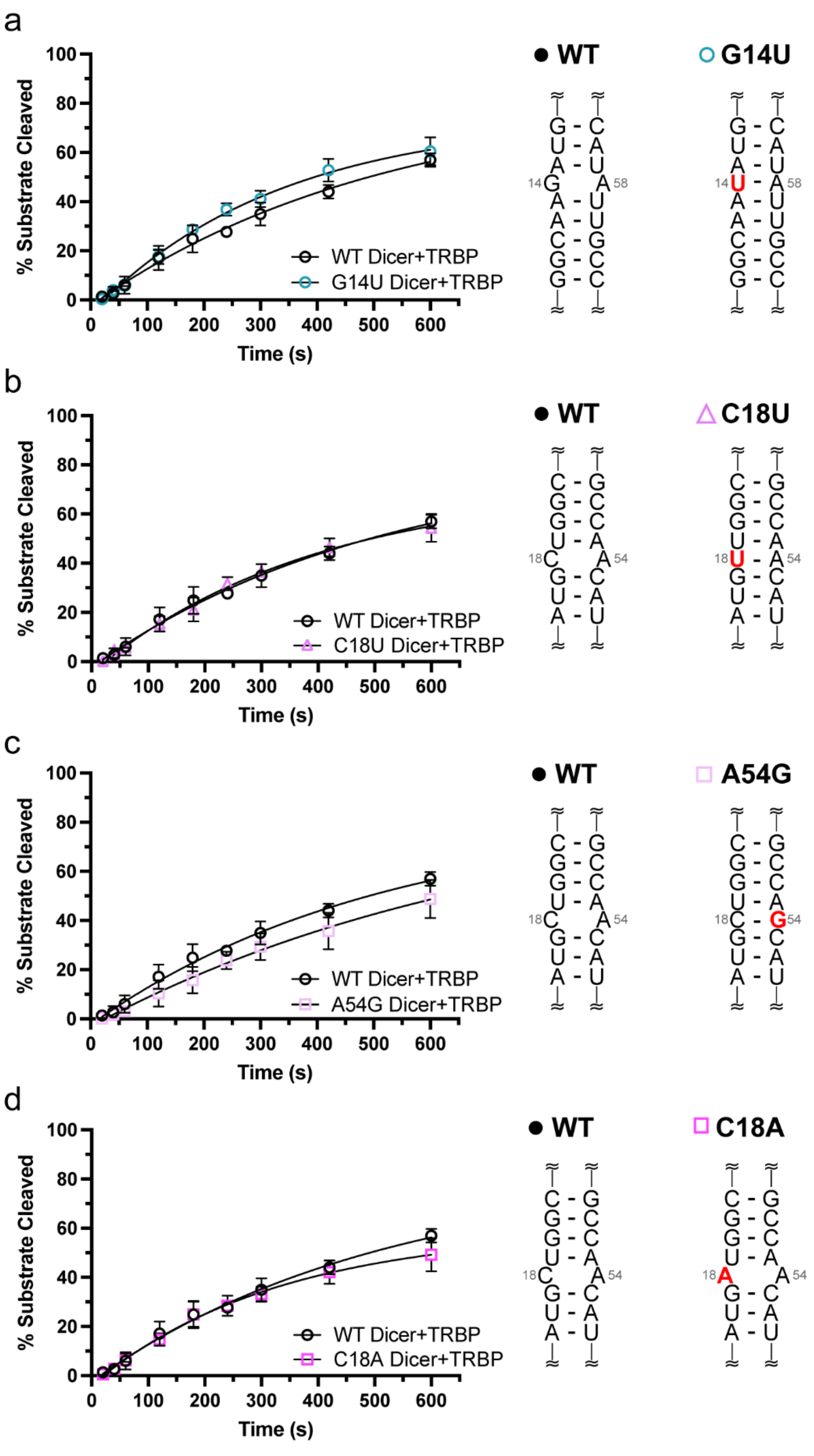

Fig. S19. Mismatches in the stem of pre-miR-31 do not significantly impact Dicer-TRBP processing of the substrate RNA. Processing assays for WT pre-miR-31 and **a)** G14U, **b)** C18U, **c)** A54G, and **d)** C18A RNAs. Quantification of pre-miR-31 processing with the Dicer-TRBP complex. Average and standard deviation from n=3 independent assays are presented. Regions of the secondary structures of constructs designed to stabilize or destabilize the stem mismatches are included for clarity. Mutations are indicated with red lettering.

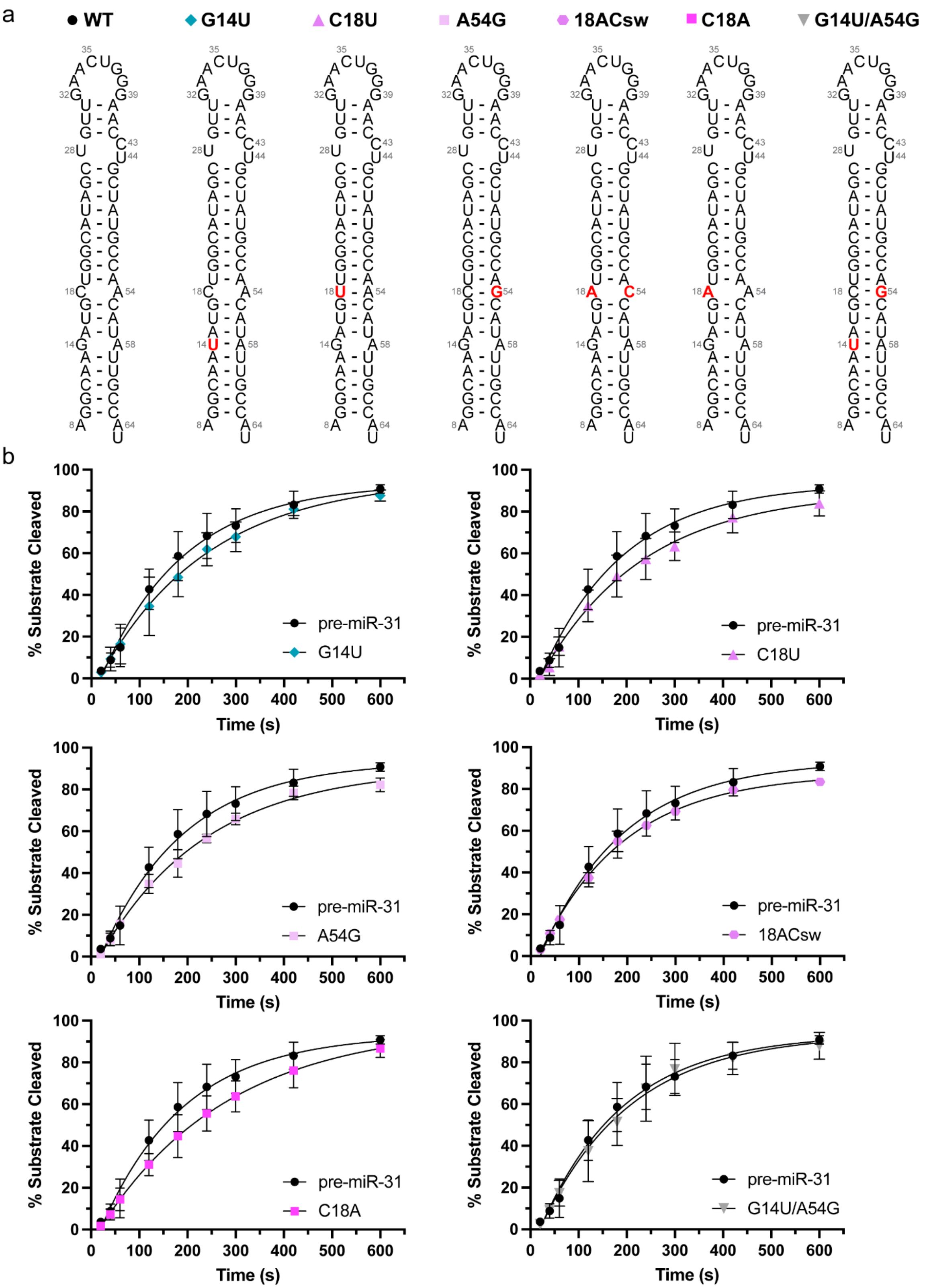

Fig. S20. Mismatches in the stem region have no impact on Dicer processing. a) Secondary structures of constructs designed to stabilize or destabilize the stem mismatches. Mutations are indicated with red lettering. **b)** Dicer processing assay of pre-miR-31 RNAs. Average and standard deviation from n=3 independent assays are presented.

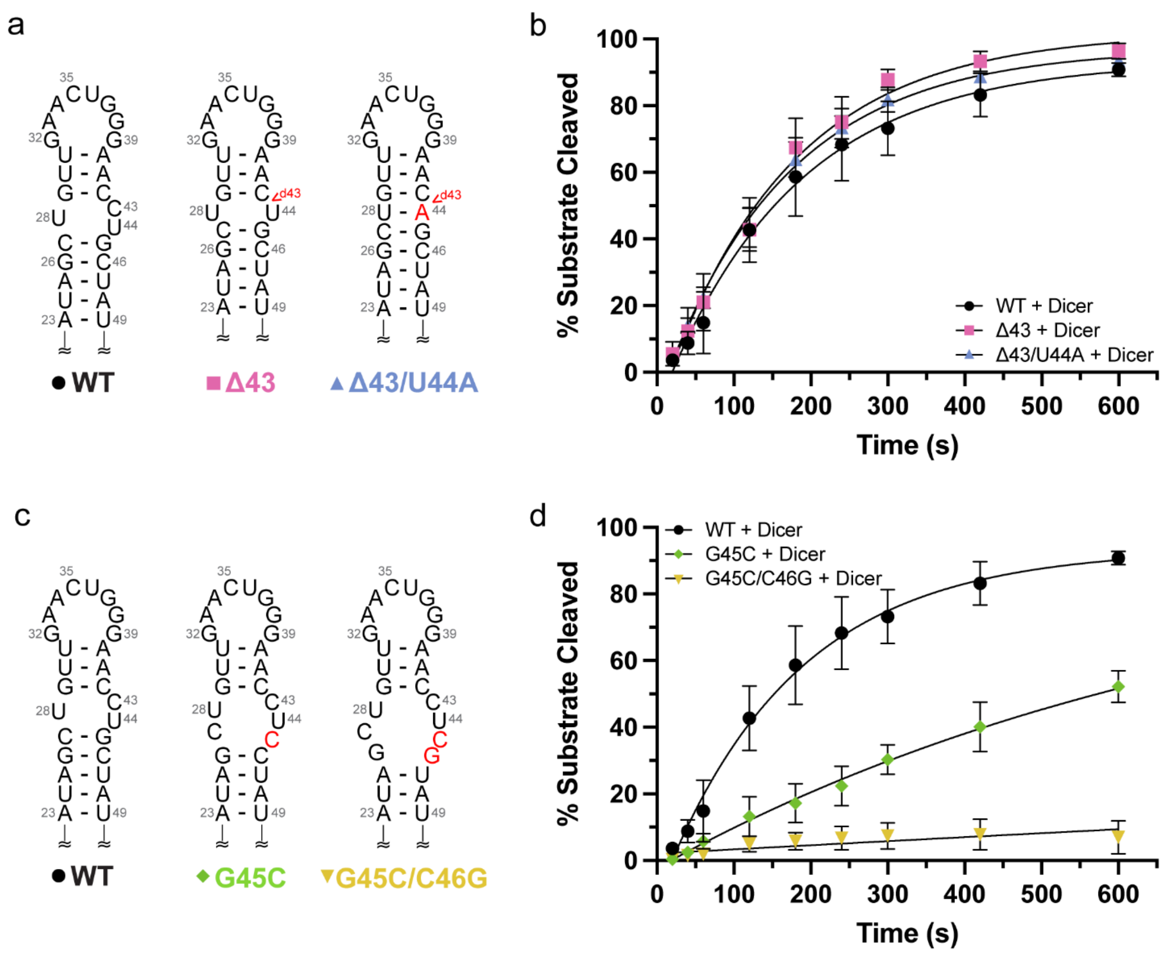

Fig. S21. Structure at the dicing site serves as a control element for Dicer processing. a) Predicted secondary structures of constructs designed to minimize the internal loop at the dicing site. Mutations are indicated with red lettering. **b)** Minimization of the internal loop at the Dicing site does not inhibit Dicer processing. **c)** Dicing site mutants with expanded internal loop structures. Mutations are indicated with red lettering. **d)** Pre-miR-31 RNAs with larger internal loops at the Dicer cleavage site have reduced Dicer processing, relative to WT.

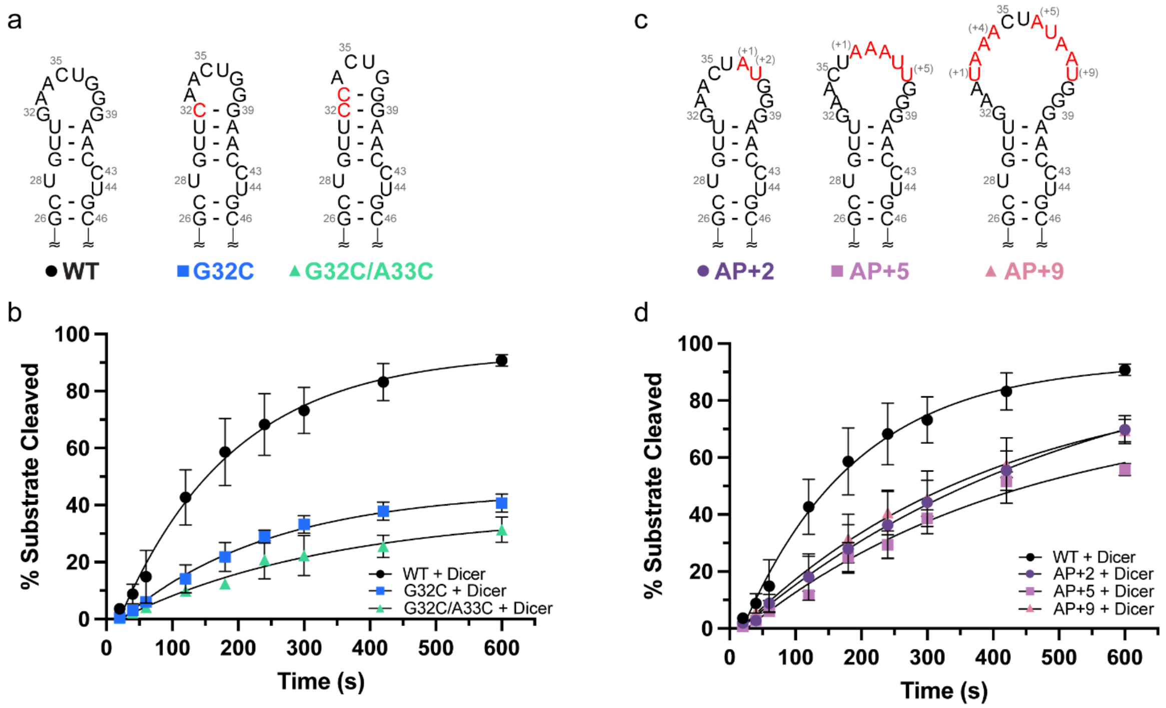

Fig. S22. Dicer processing assays for apical loop mutations. a) Secondary structures of pre-miR-31 RNAs with smaller apical loops. Sites of mutation are denoted with red lettering. **b)** *In vitro* Dicer processing assays reveal a significant reduction in substrate cleavage for G32C and G32C/A33C RNAs. **c)** Secondary structures of mutants designed to extend the pre-miR-31 apical loop. Insertions are indicated with red lettering. **d)** Dicer processing of pre-miR-31 RNAs with larger apical loops was moderately reduced relative to WT. Average and standard deviation from n=3 independent assays are presented.

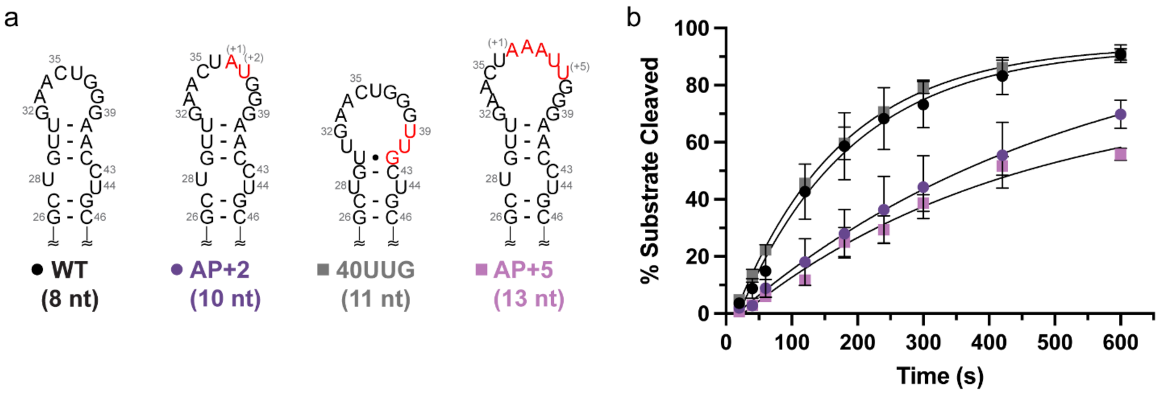

Fig. S23. A two base-pair junction between the apical loop and dicing site recovers reduced Dicer processing efficiency due to large apical loop size. a) Secondary structures of WT, AP+2, 40UUG, and AP+5 pre-miR-31 RNAs which have 8, 10, 11, and 13 nucleotide apical loops, respectively. Mutations are indicated with red lettering. **b)** Dicer processing assay of pre-miR-31 RNAs. Average and standard deviation from n=3 independent assays are presented.

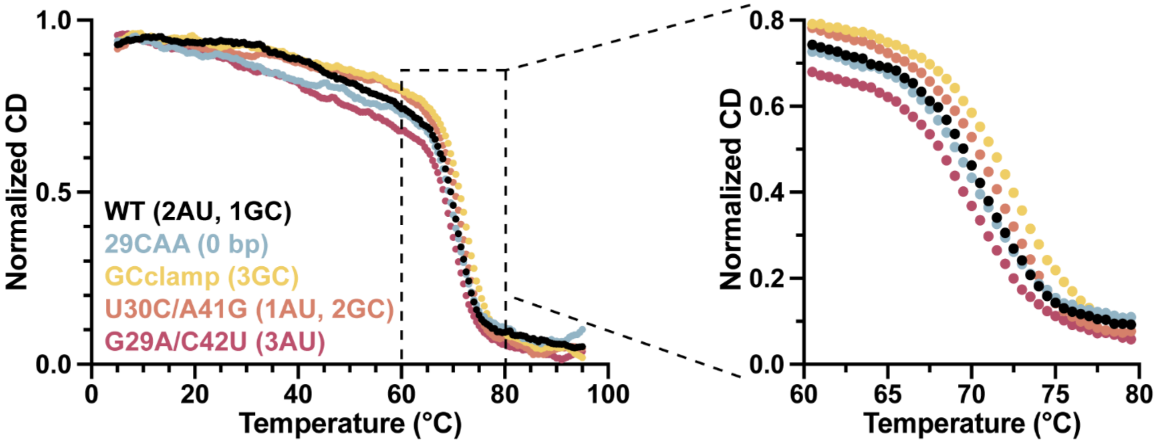

Fig. S24. Thermal stability of junction mutants. RNA thermal denaturation monitored by circular dichroism.

Table S1. Chemical shift completeness.[a]

**TopA RNA**	A (% assigned)	C (% assigned)	G (% assigned)	U (% assigned)
H8/H6	100	100	100	100
H2/H5	100	100	/	100
H1'	100	100	100	100
H2'	100	83.3	100	75
H3'	100	83.3	100	75
C6/C8	100	83.3	100	100
C2	100	/	/	/
**Top RNA**	A (% assigned)	C (% assigned)	G (% assigned)	U (% assigned)
H8/H6	100	100	90	100
H2/H5	100	100	100	100
H1'	100	100	90	100
H2'	100	100	100	100
H3'	100	100	100	100
C6/C8	100	100	90	100
C2	100	/	/	/
**FL pre-miR-31**	A (% assigned)	C (% assigned)	G (% assigned)	U (% assigned)
H8/H6	100	100	100	100
H2/H5	100	100	100	100
H1'	100	93.8	95	100
H2'	100	93.8	100	100
H3'	94.4	93.8	90	100
C8	100	/	95	/
C2	94.4	/	/	/

[a] "/" indicates a given atom is not present in the nucleoside.

Table S2. NMR restraints and structural statistics for the FL pre-miR-31 structure.[a]

**Cyana**[b]	
NOE-derived restraints	516
Intraresidue	138
Sequential	359
Long range (|i – j| > 1)	19
H-bond restraints	134
RDC	30
NOE restraints/residue	7.3
Total restraints/residue	9.6
Target function (Å2)	1.99 ± 0.02
Upper distance viol. (Å2)	0.0269 ± 0.0002
Lower distance viol. (Å2)	0.0412 ± 0.0001
RMSD[c] (Å)	2.68 ± 0.15
Q value	5.4 ± 0.3 %
**Amber**[d]	
Amber energy	-16,427.7
Distance	160.1
Torsion	7.3
RMSD[c] (Å)	2.16 ± 0.48
RMSD lower stem (1-13, 59-71) (Å)	0.22 ± 0.08
RMSD full stem (1-27, 45-71) (Å)	1.30 ± 0.37
RMSD bp in loop (29-31, 40-42) (Å)	0.33 ± 0.11
RMSD loop (32-39) (Å)	3.23 ± 1.04
**MolProbity analysis**[e]	
Clashscore	0.44
Probably wrong sugar pucker (%)	0
Bad backbone conformation (%)	4
Bad bonds (%)	0
Bad angles (%)	0

[a]Statistics are reported for the entire structure unless otherwise specified.
[b]Statistics for the 20 structures with lowest target function.
[c]RMSD: root mean squared deviation.
[d]Statistics for the 20 lowest energy structures.
[e]The 20 amber-refined structures were evaluated using the MolProbity webserver (8, 9)

Table S3. SEC-SAXS data acquisition, sample details, data analysis, model fitting, and software used.

**(*a*) Sample details**	
Organism	Human
Source	*In vitro* transcribed RNA
Scattering particle composition	
DNA/RNA(s)	Precursor microRNA 31, LM608178
Sample environment/configuration	
Solvent composition	50 mM Potassium Phosphate pH 7.5, 50 mM NaCl, 1 mM $MgCl_2$
Sample temperature (°C)	20 °C
In beam sample cell	1.0 mm ID quartz capillary
Size Exclusion Chromatography SEC-SAS	
Sample injection concentration	2.31 mg/mL
Sample injection volume	0.225 mL
SEC column type	Superdex 75 Increase 10/300 GL
SEC flowrate, mL/min	0.6 mL/min
**(*b*) SAS data collection**	
Data acquisition/reduction software	SEC-MALS-SAXS. Size separation using a Superdex 200 Increase 10/300 GL column and a 1260 Infinity II HPLC. SAXS data was measured in a sheath-flow cell(1), effective path length 0.542 mm. Data reduced with RAW 2.1.4.
Source/instrument description or reference	BioCAT facility at the Advanced Photon Source beamline 18ID with Eiger2 XE 9M (dectris) detector
Measured *q*-range ($q_{min} – q_{max}$; $Å^{-1}$, nm^{-1})	0.0027 – 0.42 $Å^{-1}$
Method for scaling intensities	Glassy Carbon, NIST SRM 3600
Exposure time(s), number of exposures. For SEC-SAS, final number of sample frames used for averaging.	0.2 s exposure time with a 1 s total exposure period (0.2 s on, 0.8 s off). 2248 exposures with 20 frames from elution peak used for averaging.
**(*c*) SAS-derived structural parameters**	
Methods/Software	Radial averaging; frame comparison, averaging, and subtraction done using BioXTAS RAW 2.1.4 (2)
Guinier Analysis	
$I(0) \pm \sigma$ ($cm^{-1;}$ a.u)	0.01 ± 3.38e-5
$R_g \pm \sigma$ (Å, nm)	28.77 ± 0.14
$min < qR_g < max$ limit (or data point range)	$0.077679 < qR_g < 11.959689$
Linear fit assessment (definition)	0.98 (r^2)
PDDF/P(r) analysis	
$I(0) \pm \sigma$ ($cm^{-1;}$ a.u.)	0.01 ± 3.9e-5 (a.u.)
$R_g \pm \sigma$ (Å, nm)	30.46 ± 0.16 (Å)
d_{max} (Å, nm)	106.0 (Å)
q-range ($Å^{-1}$, nm^{-1})	0.005 – 0.416 ($Å^{-1}$)
P(*r*) fit assessment (definition)	0.584 (χ^2)
**(*d*) Scattering particle size**	
Methods/Software	BioXTAS RAW 2.1.4 (2)
Volume estimates	
Porod volume, V_p ($Å^3$, nm^3)	25.9 ($Å^3$)
Molecular weight (M) estimates (kDa)	
From chemical composition	23.1
From SAS, concentration independent method	25.5 (Vc)

From SAS-independent measure	23.0 (MALS)
**(*e*) Modelling (a complete sub-panel for each method)**	
Shape modelling method(s) (if used)	DENSS
Software	BioXTAS RAW 2.1.4 (2)
q-range for fit ($q_{min} – q_{max}$; Å^{-1}, nm^{-1})	
Symmetry/anisotropy assumptions	
Number of individual model reconstructions	20
χ^2, CorMap *P*-values for fit	3.398e-3 (χ^2)
**(*f*) Data and model deposition**	
SASBDB IDs	SASDRF9

Table S4. Dicer+TRBP cleavage of pre-miR-31 RNAs.

Region mutated	RNA constructs	% substrate cleaved (10 min)[a]	k_{obs} (ms^{-1})[a]	k_{obs} Fold change	P value relative to WT[b]	Difference Level[c]
-	pre-miR-31 WT	57.0 ± 2.8	1.73 ± 0.17	-	-	-
Stem mutations	pre-miR-31 G14U	60.5 ± 5.7	2.09 ± 0.27	1.20	0.128	NS
	pre-miR-31 C18U	54.3 ± 5.7	1.75 ± 0.22	1.01	0.9489	NS
	pre-miR-31 A54G	48.8 ± 7.8	1.33 ± 0.30	0.77	0.1145	NS
	pre-miR-31 C18A	49.2 ± 6.8	1.58 ± 0.22	0.91	0.4004	NS
Dicing site mutations	pre-miR-31 Δ43C	80.3 ± 7.3	5.65 ± 1.25	3.26	0.0057	**
	pre-miR-31 Δ43/U44A	84.5 ± 2.5	4.70 ± 0.16	2.71	<0.0001	****
	pre-miR-31 G45C	40.1 ± 7.0	1.11 ± 0.26	0.64	0.0255	*
	pre-miR-31 G45C/C46G	31.4 ± 9.7	0.84 ± 0.18	0.49	0.0035	**
Apical loop mutations	pre-miR-31 G32C	48.3 ± 2.1	1.52 ± 0.18	0.88	0.2146	NS
	pre-miR-31 G32C/A33C	26.4 ± 5.3	0.71 ± 0.14	0.41	0.0013	**
	pre-miR-31 AP+2	52.3 ± 4.9	1.62 ± 0.13	0.93	0.4079	NS
	pre-miR-31 AP+5	62.3 ± 6.9	2.07 ± 0.33	1.20	0.1917	NS
	pre-miR-31 AP+9	44.2 ± 3.5	1.39 ± 0.17	0.80	0.0703	NS
Junction mutations	pre-miR-31 29CAA	12.3 ± 5.2	0.37 ± 0.13	0.21	0.0004	***
	pre-miR-31 GCclamp	23.5 ± 3.0	0.58 ± 0.08	0.33	0.0005	***
	pre-miR-31 U30C/A41G	59.2 ± 5.2	2.16 ± 0.18	1.25	0.0421	*
	pre-miR-31 G29A/C42U	16.4 ± 5.4	0.39 ± 0.10	0.23	0.0003	***

[a] Average and standard deviation from n=3 independent assays are presented.
[b] P value based on an unpaired parametric t-test.
[c] * P value <0.05, ** P value <0.01, *** P value <0.001, **** P value <0.0001, NS indicates no significant difference.

Table S5. Dicer cleavage of pre-miR-31 RNAs.

Region mutated	RNA constructs	% substrate cleaved (10 min)[a]	k_{obs} (ms^{-1})[a]	k_{obs} fold change	P value relative to WT[b]	Difference Level[c]
-	pre-miR-31 WT	90.8 ± 1.7	4.90 ± 1.07	-	-	-
Stem mutations	pre-miR-31 G14U	87.6 ± 2.7	4.19 ± 0.88	0.85	0.423	NS
	pre-miR-31 C18U	83.8 ± 6.1	3.82 ± 0.88	0.78	0.250	NS
	pre-miR-31 A54G	82.2 ± 3.2	3.72 ± 0.22	0.76	0.135	NS
	pre-miR-31 G14U/A54G	88.0 ± 6.4	4.71 ± 1.38	0.96	0.858	NS
	pre-miR-31 18ACsw	83.4 ± 1.4	4.27 ± 0.16	0.87	0.367	NS
	pre-miR-31 C18A	86.6 ± 4.3	3.66 ± 0.75	0.75	0.175	NS
Dicing site mutations	pre-miR-31 Δ43C	98.0 ± 2.3	6.11 ± 0.80	1.25	0.146	NS
	pre-miR-31 Δ43/U44A	93.5 ± 1.2	5.72 ± 0.61	1.17	0.311	NS
	pre-miR-31 G45C	52.2 ± 4.8	1.34 ± 0.25	0.27	0.0049	**
	pre-miR-31 G45C/C46G	7.0 ± 4.9	0.28 ± 0.12	0.06	0.0017	**
Apical loop mutations	pre-miR-31 G32C	41.8 ± 3.2	1.28 ± 0.15	0.26	0.0044	**
	pre-miR-31 G32C/A33C	31.4 ± 4.4	0.86 ± 0.15	0.17	0.0029	**
	pre-miR-31 AP+2	69.8 ± 5.0	2.14 ± 0.52	0.44	0.0158	*
	pre-miR-31 AP+5	55.8 ± 2.2	1.68 ± 0.15	0.34	0.0067	**
	pre-miR-31 AP+9	69.5 ± 3.9	2.26 ± 0.39	0.46	0.0159	*
	pre-miR-31 40UUG	91.0 ± 3.2	5.39 ± 0.12	1.10	0.473	NS
Junction mutations	pre-miR-31 29CAA	15 ± 4	0.52 ± 0.09	0.11	0.0004	***
	pre-miR-31 GCclamp	58.1 ± 6.7	1.71 ± 0.10	0.35	0.0067	**
	pre-miR-31 U30C/A41G	91.1 ± 3.4	4.12 ± 0.39	0.84	0.300	NS
	pre-miR-31 G29A/C42U	24.8 ± 1.1	0.57 ± 0.06	0.12	0.0022	**

[a] Average and standard deviation from n=3 independent assays are presented.
[b] P value based on an unpaired parametric t-test.
[c] * P value <0.05, ** P value <0.01, *** P value <0.001, NS indicates no significant difference.

Table S6. Thermal stability of pre-miR-31 RNAs.

**Region mutated**	**RNA constructs**	**T_m (°C)[a]**
-	pre-miR-31 WT	70.5 ± 0.1
Junction mutations	pre-miR-31 29CAA	70.4 ± 0.2
	pre-miR-31 3GCclamp	72.0 ± 0.2
	pre-miR-31 U30C A41G	71.4 ± 0.2
	pre-miR-31 G29A C42U	70.3 ± 0.4

[a] T_m values were obtained by fitting CD thermal denaturation profiles to a two-state unfolding model using sloping baselines. Average and standard deviation from n=3 independent assays are presented.

Table S7. Synthetic DNA templates and associated RNA constructs.

	5'-sequence-3'[a,b,c]	
	**DNA**	**RNA**
Top	mGmGCATAGCAGGTTCCCAGTTCAACAGCTATGCC*TATAGTGAGTCGTATTA*	GGCAUAGCUGUUGAACUGGGAACCUGCUAUGCC
TopA	mGmGCATAGCCGTAGCTATGCC*TATAGTGAGTCGTATTA*	GGCAUAGCUACGGCUAUGCC

[a] m denotes 2′-*O*-Me modification of the primer.
[b] Italicized nucleotides correspond to the sequence complementary to the T7 promoter.
[c] Red nucleotides indicate non-native tetraloop sequences.

Table S8. DNA primers for pre-miR-31-tail (DMS) experiments.

OE-PCR primers	5'-sequence-3'
miR31_tail-1F	GCAGCTGAATTCTTCTAATACGACTCACTATAGGAGACCTCGAGTAG AGGTCAAAA
miR31_tail-2R	CCAGCATCTTGCCTCCTCTCCTTTTGACCTCTACTCGAGGTCTCCTAT AGTG
miR31_tail-3F	GGAGGCAAGATGCTGGCATAGCTGTTGAACTGGGAACCTGCTATGC CAACAT
miR31_tail-4R	AGTTGTTTGGAAAGATGGCAATATGTTGGCATAGCAGGTTCCC
miR31_tail-5F	TTGCCATCTTTCCAAACAACTCGAGTAGAGTTGACAACAAAGAAAC AACAACAACAACGG
miR31_tail-6R	GCAGGAGGATCCGTTGTTGTTGTTGTTTCTTTGTTGTC
miR_tail_RT	GTTGTTGTTGTTGTTTCTTTGTTGTCAACTCTACTCGAGTTGTTT
miR31_buffer_F	GGAGACCTCGAGTAGAGGTCAAAAGGAGAGG

Table S9. DNA primers for generation of the pre-miR-31 (NMR) template.

OE-PCR primers	5'-sequence-3'
miR-31FL-OE-1F	GTGTCAGAATTCTAATACGACTCACTATAGGAGAGGAGGCAAG
miR-31FL-OE-2R	CCAGTTCAACAGCTATGCCAGCATCTTGCCTCCTCTCCTATAGTGAGTCGTA
miR-31FL-OE-3F	GCTGGCATAGCTGTTGAACTGGGAACCTGCTATGCCAACATATTGCCATCTTT
miR-31FL-OE-4R	CATAGCGGATCCGGAAAGATGGCAATATGTTGGCATAGCAGGT

Table S10. Amplification primers for template.

Amplification primers	5'-sequence-3'[a]	application
UNIV-pUC19_E105	TCTTCGCTATTACGCCAGCTGGCGAAA	Forward primer for amplification of DNA template for pre-miR-31 NMR construct and all processing constructs
HDV-AMP-R	mUmAATGTGAGAATTGGCTACGTTGAAACAACGCATTACCG	Reverse primer for amplification of DNA template for all pre-miR-31 processing constructs
miR31_4R	mGmGAAAGATGGCAATATGTTGGCATAGCAGGTT	Reverse primer for amplification of DNA template for pre-miR-31 NMR construct
miR_tail_3buffer_REV	mGmUTGTTGTTGTTGTTTCTTTGTTGTCAACTCTACTCGAGTTG	Reverse primer for amplification of DNA template for pre-miR-31 DMS construct

[a] m denotes 2′-*O*-Me modification of the primer.

Table S11. DNA primers for HH-pre-miR-31 template.

OE-PCR primers	5'-sequence-3'
HH_miR31_Nat_1F	CCGGAATTCTAATACGACTCACTATAGGGCTC
HH_miR31_Nat_2R	ACGTACCCTGATGGTGTACGAGCCCTATAGTGAGTCGTATTA
HH_miR31_Nat_3F	ACACCATCAGGGTACGTTTTTCAGACACCATCAGGGTCTGGCATCTTG CCTCT
HH_miR31_Nat_4R	CTGACGGTACCGGGTACCGTTTCGTCCTCACGGACTCATCAGAGGCA AGATGCCAGACC
HH_miR31_Nat_5F	ACCCGGTACCGTCAGGCAAGATGCTGGCATAGCTGTTGAACTGGGAA CCTGCTATGCCAA
HH_miR31_Nat_6R	CCGTCGCGGATCCATGGCAATATGTTGGCATAGCAGGTTCCCAGT

Table S12. Mutation DNA primers for processing constructs.

Mutagenesis primers	5'-sequence-3'	Application
HH-miR-31-HDV-mut-F	TAATGCGTTGTTTCAACGTAGCCAATTC TCACATTAGGATCCTCTAGAGTCGAC	Forward primer to insert HDV-like sequence to 3' end of HH-pre-miR-31
HH-miR-31-HDV-mut-R	CCGACACTACGACGGGGACGTTTCTCAC TCAGTGTCATGGCAATATGTTGGCATAG	Reverse primer to insert HDV-like sequence to 3' end of HH-pre-miR-31
HH-A54G-HDV-mut-R	CCGACACTACGACGGGGACGTTTCTCAC TCAGTGTCATGGCAATATGCTGGCATAG	Reverse primer to insert HDV-like sequence to 3' end of HH-pre-miR-31-A54G
miR-31-Nat-G14U-Mut-F	CGTCAGGCAATATGCTGGCATAG	Forward primer for G14U construct mutation
miR-31-Nat-G14U-Mut-R	GTACCGGGTACCGTTTCG	Reverse primer for G14U construct mutation
miR31-G14U-11nt-F	GGTCTGGCATATTGCCTCTGA	Forward primer for G14U hammerhead complementary sequence mutation
miR31-G14U-11nt-R	CTGATGGTGTCTGAAAAACG	Reverse primer for G14U hammerhead complementary sequence mutation
miR-31-Nat-C18U-Mut-F	AGGCAAGATGTTGGCATAGCTGTTG	Forward primer for C18U construct mutation
miR-31-Nat-C18U-Mut-R	GACGGTACCGGGTACCGT	Reverse primer for C18U construct mutation
miR31-C18U-11nt-F	TCAGGGTCTGACATCTTGCCT	Forward primer for C18U hammerhead complementary sequence mutation
miR31-C18U-11nt-R	TGGTGTCTGAAAAACGTAC	Reverse primer for C18U hammerhead complementary sequence mutation
miR-31-Nat-C18A-31-F	AGGCAAGATGATGGCATAGCTG	Forward primer for C18A construct mutation
miR-31-Nat-C18A-31-R	GACGGTACCGGGTACCGT	Reverse primer for C18A construct mutation
miR-31-Nat-C18A-11nt-F	TCAGGGTCTGTCATCTTGCCT	Forward primer for C18A hammerhead complementary sequence mutation
miR-31-Nat-C18A-11nt-R	TGGTGTCTGAAAAACGTAC	Reverse primer for C18A hammerhead complementary sequence mutation
miR31-18ACsw-mut-F	TGCTATGCCACCATATTGCCATG	Forward primer for 18Acsw construct mutation
miR31-18ACsw-mut-R	GGTTCCCAGTTCAACAGC	Reverse primer for 18Acsw construct mutation
miR-31-Nat-29CAA-Mut-F	TGGCATAGCTCAAGAACTGGGAACC	Forward primer for 29CAA construct mutation
miR-31-Nat-29CAA-Mut-R	GCATCTTGCCTGACGGTA	Reverse primer for 29CAA construct mutation
miR31-nat-G32C-F	CATAGCTGTTCAACTGGGAACC	Forward primer for G32C construct mutation
miR31-nat-G32C-R	CCAGCATCTTGCCTGACG	Reverse primer for G32C construct mutation
miR31-Nat-G32C/A33C-F	CATAGCTGTTCCACTGGGAACCTG	Forward primer for G32C/A33C construct mutation

miR31-Nat-G32C/A33C-R	CCAGCATCTTGCCTGACG	Reverse primer for G32C/A33C construct mutation
miR31-nat-40UUG-mut-F	TTGAACTGGGTTGCTGCTATGCCAAC	Forward primer for 40UUG construct mutation
miR31-nat-40UUG-mut-R	CAGCTATGCCAGCATCTTG	Reverse primer for 40UUG construct mutation
miR31-nat-A54G-mut-F	TGCTATGCCAGCATATTGCCAT	Forward primer for A54G construct mutation
miR31-nat-A54G-mut-R	GGTTCCCAGTTCAACAGC	Reverse primer for A54G construct mutation
miR31-AP+2-mut-F	ATGGGAACCTGCTATGCCAA	Forward primer for AP+2 construct mutation
miR31-AP+2-mut-R	AGTTCAACAGCTATGCCAG	Reverse primer for AP+2 construct mutation
miR31-AP+5-mut-F	ATAATGGGAACCTGCTATGCCAA	Forward primer for AP+5 construct mutation
miR31-AP+5-mut-R	AGTTCAACAGCTATGCCAG	Reverse primer for AP+5 construct mutation
miR31-AP+9-mut-F	TATAATGGGAACCTGCTATGCCAA	Forward primer for AP+9 construct mutation
miR31-AP+9-mut-R	GTTTATTCAACAGCTATGCCAGCAT	Reverse primer for AP+9 construct mutation
miR31-3Gcclamp-mut-F	TGGGCGCCTGCTATGCCAACATATTG	Forward primer for 3Gcclamp construct mutation
miR31-3Gcclamp-mut-R	GTTCCGCAGCTATGCCAGCATCTTG	Reverse primer for 3Gcclamp construct mutation
miR31-G45C-mut-F	CTGGGAACCTCCTATGCCAAC	Forward primer for G45C construct mutation
miR31-G45C-mut-R	TTCAACAGCTATGCCAGC	Reverse primer for G45C construct mutation
miR31-G45C C46G-mut-F	CTGGGAACCTCGTATGCCAACATATTG	Forward primer for G45C/C46G construct mutation
miR31-G45C C46G-mut-R	TTCAACAGCTATGCCAGC	Reverse primer for G45C/C46G construct mutation
miR31-d43C-mut-F	TGCTATGCCAACATATTGC	Forward primer for Δ43 construct mutation
miR31-d43C-mut-R	GTTCCCAGTTCAACAGCTATG	Reverse primer for Δ43 construct mutation
miR31-4344A-mut-F	AACTGGGAACAGCTATGCCAAC	Forward primer for Δ43/U44A construct mutation
miR31-4344A-mut-R	CAACAGCTATGCCAGCAT	Reverse primer for Δ43/U44A construct mutation
G29A C42U-mut-FWD	TGGGAATCTGCTATGCCAACATATTG	Forward primer for G29A/C42U construct mutation
G29A C42U-mut-REV	GTTCAATAGCTATGCCAGCATCTTG	Reverse primer for G29A/C42U construct mutation
U30C A41G-mut-FWD	TGGGAGCCTGCTATGCCAACATATTG	Forward primer for U30C/A41G construct mutation
U30C A41G-mut-REV	GTTCAGCAGCTATGCCAGCATCTTG	Reverse primer for U30C/A41G construct mutation

Table S13. RNA sequences used for structural and processing studies.

construct name	5'-RNA sequence-3'	Application
miR-31_DMS	GGAGACCUCGAGUAGAGGUCAAAAGGAGAGGAGGC AAGAUGCUGGCAUAGCUGUUGAACUGGGAACCUGC UAUGCCAACAUAUUGCCAUCUUUCCAAACAACUCGA GUAGAGUUGACAACAAAGAAACAACAACAACAAC	DMS chemical probing
FL-pre-miR-31	GGAGAGGAGGCAAGAUGCUGGCAUAGCUGUUGAAC UGGGAACCUGCUAUGCCAACAUAUUGCCAUCUUUCC	Structure
WT pre-miR-31	AGGCAAGAUGCUGGCAUAGCUGUUGAACUGGGAAC CUGCUAUGCCAACAUAUUGCCAU	Processing
WT pre-miR-31 -G14U	AGGCAAUAUGCUGGCAUAGCUGUUGAACUGGGAAC CUGCUAUGCCAACAUAUUGCCAU	Processing
WT pre-miR-31-C18U	AGGCAAGAUGUUGGCAUAGCUGUUGAACUGGGAAC CUGCUAUGCCAACAUAUUGCCAU	Processing
WT pre-miR-31-C18A	AGGCAAGAUGAUGGCAUAGCUGUUGAACUGGGAAC CUGCUAUGCCAACAUAUUGCCAU	Processing
WT pre-miR-31-18Acsw	AGGCAAGAUGAUGGCAUAGCUGUUGAACUGGGAAC CUGCUAUGCCACCAUAUUGCCAU	Processing
WT pre-miR-31-A54G	AGGCAAGAUGCUGGCAUAGCUGUUGAACUGGGAAC CUGCUAUGCCAGCAUAUUGCCAU	Processing
WT pre-miR-31-40UUG	AGGCAAGAUGCUGGCAUAGCUGUUGAACUGGGUUG CUGCUAUGCCAACAUAUUGCCAU	Processing
WT pre-miR-31-29CAA	AGGCAAGAUGCUGGCAUAGCUCAAGAACUGGGAAC CUGCUAUGCCAACAUAUUGCCAU	Processing
WT pre-miR-31-G32C	AGGCAAGAUGCUGGCAUAGCUGUUCAACUGGGAAC CUGCUAUGCCAACAUAUUGCCAU	Processing
WT pre-miR-31-G32C/A33C	AGGCAAGAUGCUGGCAUAGCUGUUCCACUGGGAAC CUGCUAUGCCAACAUAUUGCCAU	Processing
WT pre-miR-31-G14U/A54G	AGGCAAUAUGCUGGCAUAGCUGUUGAACUGGGAAC CUGCUAUGCCAGCAUAUUGCCAU	Processing
WT pre-miR-31-AP+2	AGGCAAGAUGCUGGCAUAGCUGUUGAACUAUGGGA ACCUGCUAUGCCAACAUAUUGCCAU	Processing
WT pre-miR-31-AP+5	AGGCAAGAUGCUGGCAUAGCUGUUGAACUAUAAUG GGAACCUGCUAUGCCAACAUAUUGCCAU	Processing
WT pre-miR-31-AP+9	AGGCAAGAUGCUGGCAUAGCUGUUGAAUAAACUAU AAUGGGAACCUGCUAUGCCAACAUAUUGCCAU	Processing
WT -miR-31-Gcclamp	AGGCAAGAUGCUGGCAUAGCUGCGGAACUGGGCGC CUGCUAUGCCAACAUAUUGCCAU	Processing
WT pre-miR-31-G45C	AGGCAAGAUGCUGGCAUAGCUGUUGAACUGGGAAC CUCCUAUGCCAACAUAUUGCCAU	Processing
WT pre-miR-31-G45C/C46G	AGGCAAGAUGCUGGCAUAGCUGUUGAACUGGGAAC CUCGUAUGCCAACAUAUUGCCAU	Processing
WT pre-miR-31-Δ43	AGGCAAGAUGCUGGCAUAGCUGUUGAACUGGGAAC UGCUAUGCCAACAUAUUGCCAU	Processing
WT pre-miR-31- Δ43/U44A	AGGCAAGAUGCUGGCAUAGCUGUUGAACUGGGAAC AGCUAUGCCAACAUAUUGCCAU	Processing
WT pre-miR-31-U30C/A41G	AGGCAAGAUGCUGGCAUAGCUGCUGAACUGGGAGC CUGCUAUGCCAACAUAUUGCCAU	Processing
WT pre-miR-31-G29A/C42U	AGGCAAGAUGCUGGCAUAGCUAUUGAACUGGGAAU CUGCUAUGCCAACAUAUUGCCAU	Processing

SI References

1. N. Kirby *et al.*, Improved radiation dose efficiency in solution SAXS using a sheath flow sample environment. *Acta Crystallographica Section D: Structural Biology* **72**, 1254-1266 (2016).
2. J. B. Hopkins, R. E. Gillilan, S. Skou, BioXTAS RAW: improvements to a free open-source program for small-angle X-ray scattering data reduction and analysis. *Journal of applied crystallography* **50**, 1545-1553 (2017).
3. D. Svergun, Determination of the regularization parameter in indirect-transform methods using perceptual criteria. *Journal of applied crystallography* **25**, 495-503 (1992).
4. D. Schneidman-Duhovny, M. Hammel, J. A. Tainer, A. Sali, FoXS, FoXSDock and MultiFoXS: Single-state and multi-state structural modeling of proteins and their complexes based on SAXS profiles. *Nucleic acids research* **44**, W424-W429 (2016).
5. D. Schneidman-Duhovny, M. Hammel, J. A. Tainer, A. Sali, Accurate SAXS profile computation and its assessment by contrast variation experiments. *Biophysical journal* **105**, 962-974 (2013).
6. P. Z. Johnson, W. K. Kasprzak, B. A. Shapiro, A. E. Simon, RNA2Drawer: geometrically strict drawing of nucleic acid structures with graphical structure editing and highlighting of complementary subsequences. *RNA biology* **16**, 1667-1671 (2019).
7. C. Hartlmueller *et al.*, RNA structure refinement using NMR solvent accessibility data. *Scientific reports* **7**, 1-10 (2017).
8. D. I. L.-F. A. Chen, V. B. J. K. G. Wang, X. MolProbity: all-atom contacts and structure validation for proteins and nucleic acids. *Nucleic Acids Res* **35**, W375-W383 (2007).
9. C. J. Williams *et al.*, MolProbity: More and better reference data for improved all-atom structure validation. *Protein Science* **27**, 293-315 (2018).
